# Optimum population-level use of artemisinin combination therapies: a modelling study

**DOI:** 10.1016/S2214-109X(15)00162-X

**Published:** 2015-11-04

**Authors:** Tran Dang Nguyen, Piero Olliaro, Arjen M Dondorp, J Kevin Baird, Ha Minh Lam, Jeremy Farrar, Guy E Thwaites, Nicholas J White, Maciej F Boni

**Affiliations:** a Oxford University Clinical Research Unit, Wellcome Trust Major Overseas Programme, Ho Chi Minh City, Vietnam; b University of Oxford, Oxford, UK; c Mahidol-Oxford Research Unit, Wellcome Trust Major Overseas Programme, Bangkok, Thailand; d Eikman-Oxford Clinical Research Unit, Jakarta, Indonesia; e Wellcome Trust, London, UK

## Abstract

**Background:**

Artemisinin combination therapies (ACTs) are used worldwide as first-line treatment against confirmed or suspected *Plasmodium falciparum* malaria. Despite the success of ACTs at reducing the global burden of malaria, emerging resistance to artemisinin threatens these gains. Countering onset of resistance might need deliberate tactics aimed at slowing the reduction in ACT effectiveness. We assessed optimum use of ACTs at the population level, specifically focusing on a strategy of multiple first-line therapies (MFT), and comparing it with strategies of cycling or sequential use of single first-line ACTs.

**Methods:**

With an individual-based microsimulation of regional malaria transmission, we looked at how to apply a therapy as widely as possible without accelerating reduction of efficacy by drug resistance. We compared simultaneous distribution of artemether–lumefantrine, artesunate–amodiaquine, and dihydroartemisinin–piperaquine (ie, MFT) against strategies in which these ACTs would be cycled or used sequentially, either on a fixed schedule or when population-level efficacy reaches the WHO threshold of 10% treatment failure. The main assessment criterion was total number of treatment failures per 100 people per year. Additionally, we analysed the benefits of including a single non-ACT therapy in an MFT strategy, and did sensitivity analyses in which we varied transmission setting, treatment coverage, partner-drug half-life, fitness cost of drug resistance, and the relation between drug concentration and resistance evolution.

**Findings:**

Use of MFT was predicted to reduce the long-term number of treatment failures compared with strategies in which a single first-line ACT is recommended. This result was robust to various epidemiological, pharmacological, and evolutionary features of malaria transmission. Inclusion of a single non-ACT therapy in an MFT strategy would have substantial benefits in reduction of pressure on artemisinin resistance evolution, delaying its emergence and slowing its spread.

**Interpretation:**

Adjusting national antimalarial treatment guidelines to encourage simultaneous use of MFT is likely to extend the useful therapeutic life of available antimalarial drugs, resulting in long-term beneficial outcomes for patients.

**Funding:**

Wellcome Trust, UK Medical Research Council, Li Ka Shing Foundation.

## Introduction

During the next decade, substantial public health effort and financial resources will be expended to eliminate malaria in as many parts of the world as possible.^1^ In this endeavour, several antimalarial strategies will be used, including antimalarial treatment for clinically acute cases, antimalarial chemoprophylaxis in at-risk populations, insecticide-treated nets, household insecticide use, improved diagnostics, and expansion of health service delivery.^2^ For some of these interventions, long-term diminishing returns as drug or insecticide resistance emerges and as mosquitoes adapt their behaviour to insecticide-treated nets and insecticide application are of concern. Efforts to maximise effects of elimination campaigns and to minimise the chances of an emergent drug or insecticide resistance phenomenon occurring during this period are important.^3^ Such an event would seriously undermine the ambition and progress of malaria elimination programmes.

Since 2005, WHO has strongly endorsed first-line use of artemisinin-based combination therapies (ACTs) for uncomplicated *Plasmodium falciparum* malaria because of their safety and rapid action against asexual blood stages, including some transmission stages.^4^, ^5^ Additionally, WHO discouraged artemisinin monotherapy to reduce recrudescence rates and to decrease the probability of de-novo artemisinin resistance emerging in individual patients. The partner drug in a coformulated ACT is always eliminated more slowly than the rapidly eliminated artemisinin derivatives, providing protection during the course of treatment against de-novo emergence of an artemisinin-resistant genotype. For artemisinin resistance to emerge, a parasite must be capable of surviving exposure to artemisinin and the partner drug—a highly improbable event unless the infecting parasite population already carries resistance genes to the partner drug.

Despite these precautions aimed at preserving efficacy of artemisinin-based therapies, artemisinin resistance still warrants serious concern. A partly resistant, slow-clearing *P falciparum* phenotype emerged in Cambodia in the 1980s or 1990s.^6^, ^7^ This phenotype was later seen on the border between Thailand and Myanmar^8^ and in southern Vietnam near the Cambodian border,^9^, ^10^ and is now established in much of mainland southeast Asia.^10^, ^11^ Slow parasite clearance is strongly associated with a group of polymorphisms in the *P falciparum* kelch propeller domain.^6^, ^10^ When devising strategies to contain or extinguish this resistant genotype, the potential for stronger resistance and emergence of a fully artemisinin-resistant genotype in the next decade should also be considered.

Panel **Research in context**

**Evidence before this study**

We searched PubMed combining the term “malaria OR plasmodium OR falciparum” with “treatment strategy” (22 results), “resistance management” (77 results), “multiple first” (seven results), “multiple drugs” (22 results), “two drugs” (92 results), and “drug distribution” (25 results), which yielded three publications in which mathematical models were used to compare different methods of distributing several available antimalarial therapies.

**Added value of this study**

The model presented here is an advance over these studies because it includes individual-level host detail, age-specific effects, explicit tracking of within-host parasite density, and drug-specific pharmacodynamics. Additionally, we validated the present model's behaviour against a range of field and clinical datasets to ensure that the model replicates well known clinical and epidemiological patterns of *Plasmodium falciparum* malaria.

**Implications of all the available evidence**

At national or regional levels, simultaneous use of multiple first-line artemisinin combination therapies should be recommended, because this approach will delay emergence and evolution of artemisinin-resistant parasites and partner-drug-resistant parasites.

Artemisinin pressure on parasites is likely to increase during the next decade for the following reasons: ACTs will remain the most commonly used antimalarial therapies; existing partner-drug resistance is likely to spread to areas where the corresponding ACTs are used; and artemisinin monotherapies are still likely to be used to some extent in contravention of strong WHO and national-level health policy recommendations. Additionally, in individual patients, underdosing with artemisinin-based drugs might be a concern because subtherapeutic doses create an environment favourable to fixation of drug-resistant genotypes.^12^ Underdosing might occur as a result of substandard drugs,^13^ insufficient absorption, poor adherence practices, or prescription of subtherapeutic doses, especially in hyperparasitaemic patients (who need higher doses than patients with lower parasite densities) and young children or pregnant women (who have low drug exposures).^12^, ^14^ For these reasons, additional measures should be taken to ensure that the evolutionary selection pressure for artemisinin-resistant genotypes is as low as possible for as long as possible.

A key biological principle underpinning potential strategies for slowing down evolution and spread of a novel mutant is that evolution occurs slowly in heterogeneous or variable environments.^15^ Combination therapy takes advantage of this principle by introducing drug heterogeneity into a pathogen's environment and forcing the pathogen population to adapt to several new environmental features simultaneously. This principle can be applied at the population level, if a parasite encounters different drugs in different individuals. The two frequently explored approaches to achieve this effect are drug cycling—in which a single therapy is used population-wide for a specific amount of time before being replaced with a different therapy—and simultaneous distribution of several therapies in a population. Both strategies have been assessed with mathematical models for bacteria^16^, ^17^, ^18^ and malaria,^19^, ^20^, ^21^, ^22^ and simultaneous distribution is generally more effective than drug cycling at delaying resistance evolution and keeping prevalence low for a longer period. One of the reasons is that, with a strategy of simultaneous distribution of different drugs, the parasite's environment is more variable than with a cycling strategy.^18^, ^19^ In this scenario, even if a de-novo resistant parasite were to emerge in a single host, it would have difficulty establishing itself in the population because the parasite's next host would have at least a 50% chance of not being treated with the same drug. This effect would be as strong in a cycling strategy only if the drugs were cycled in and out rapidly, on the order of the generation time of the infection.

We assessed optimum distribution of ACTs at the population level, specifically focusing on a strategy of multiple first-line therapies (MFT)^19^—in which therapies are simultaneously recommended as first-line and are prescribed to individual patients according to a random factor (eg, day of week or true randomisation)—and comparing it with strategies of cycling or sequential use of single first-line ACTs. We developed and validated an individual-based microsimulation, which is an advance over previous efforts to address this question because it accounts for key features of malaria epidemiology that affect patterns of resistance evolution: age-specific immune acquisition, biting rate heterogeneity, drug pharmacokinetics and pharmacodynamics, asexual parasite density, multiplicity of infection, and recombination.

## Methods

### Study design

We developed an individual-based stochastic microsimulation of *P falciparum* transmission (appendix). The model tracks 1 million individuals for 20 years, and assesses which population-level approaches to distribution of ACTs most effectively reduce the cumulative number of treatment failures over 20 years and minimise the risk of emergence of a novel artemisinin-resistant genotype.

### Validation

For some model parameters, such as duration of infection or parasite density levels, we obtained direct measurements from field or clinical data and input them into the model (appendix). When direct measurements were not available—eg, for age-specific rates of immune acquisition—we validated the model by comparing model outputs with the corresponding field observations (appendix). When a behaviour could vary greatly—eg, the relation between drug concentration and probability that a drug-resistant mutant will emerge—we assessed several possibilities in a sensitivity analysis.

### Strategy comparison and assessment criteria

We assumed that three ACTs with different partner drugs were available for treatment. We set half-lives used for the three partner drugs to 4·5 days for lumefantrine, 9 days for amodiaquine, and 28 days for piperaquine.^23^, ^24^, ^25^, ^26^ We compared three population-level treatment strategies in the simulations. The first strategy is use of MFT, in which a third of individuals are treated with artemether–lumefantrine, a third with artesunate–amodiaquine, and a third with dihydroartemisinin–piperaquine. The second strategy is a cycling strategy, in which a single ACT is used in the population at any one time and the ACTs are rotated on a 5-year schedule; shorter half-life ACTs are rotated in first, so the sequence is artemether–lumefantrine, artesunate–amodiaquine, then dihydroartemisinin–piperaquine. The third strategy mimics the standard approach of cycling or sequentially using first-line therapies until they begin to fail. This strategy is called sequential deployment, and therapies are replaced when the treatment failure rate (with a 60-day moving average) reaches the WHO-defined^4^ criterion of 10%; a 1-year delay is built in to this strategy, because switching first-line drugs at the national level usually takes longer than 1 year.^27^

We used four assessment criteria. The total number of treatment failures per 100 people per year, discounted at 3% annually, is the main assessment criterion for comparing strategies; non-treatments are counted as treatment failures in this calculation. The useful therapeutic life of a strategy is defined as the amount of time a strategy can be used before we see 10% treatment failure population-wide; for comparison, in the cycling and sequential deployment strategies, we count the total time in the simulation that the treatment failure rate is below 10% and define this as the useful therapeutic life. We define time until resistance emergence (*T*_0·01_) as the time at which the genotype frequency of all resistant alleles (appendix) reaches 1%, and we used this as an approximate threshold of when resistant genotypes progress from rare to nearly established. Finally, we monitored artemisinin monotherapy use; we define de-facto artemisinin monotherapy use as ACT treatment of an infection with partner-drug resistance. We report the total number of individual cases of artemisinin monotherapy use in a 20-year simulation, which measures how exposed (ie, not protected by a partner drug) artemisinin drugs are to resistance evolution.

### Statistical analysis

When comparing two strategies, we tested statistically for rank differences in the outcome measures (Mann-Whitney test) and differences between the medians and IQR (Mood's test). To summarise, we present the maximum of these four p values, unless stated otherwise.

We did sensitivity analyses to identify effects of differences in transmission setting, treatment coverage, partner-drug half-life, fitness cost of drug resistance, and the relation between drug concentration and resistance evolution (appendix).

### Role of the funding source

The funders of the study had no role in model design, analysis, interpretation, or writing of the report. The corresponding author had full access to all model code, validation results, simulation results, and had final responsibility for the decision to submit for publication.

## Results

**Figure 1:**
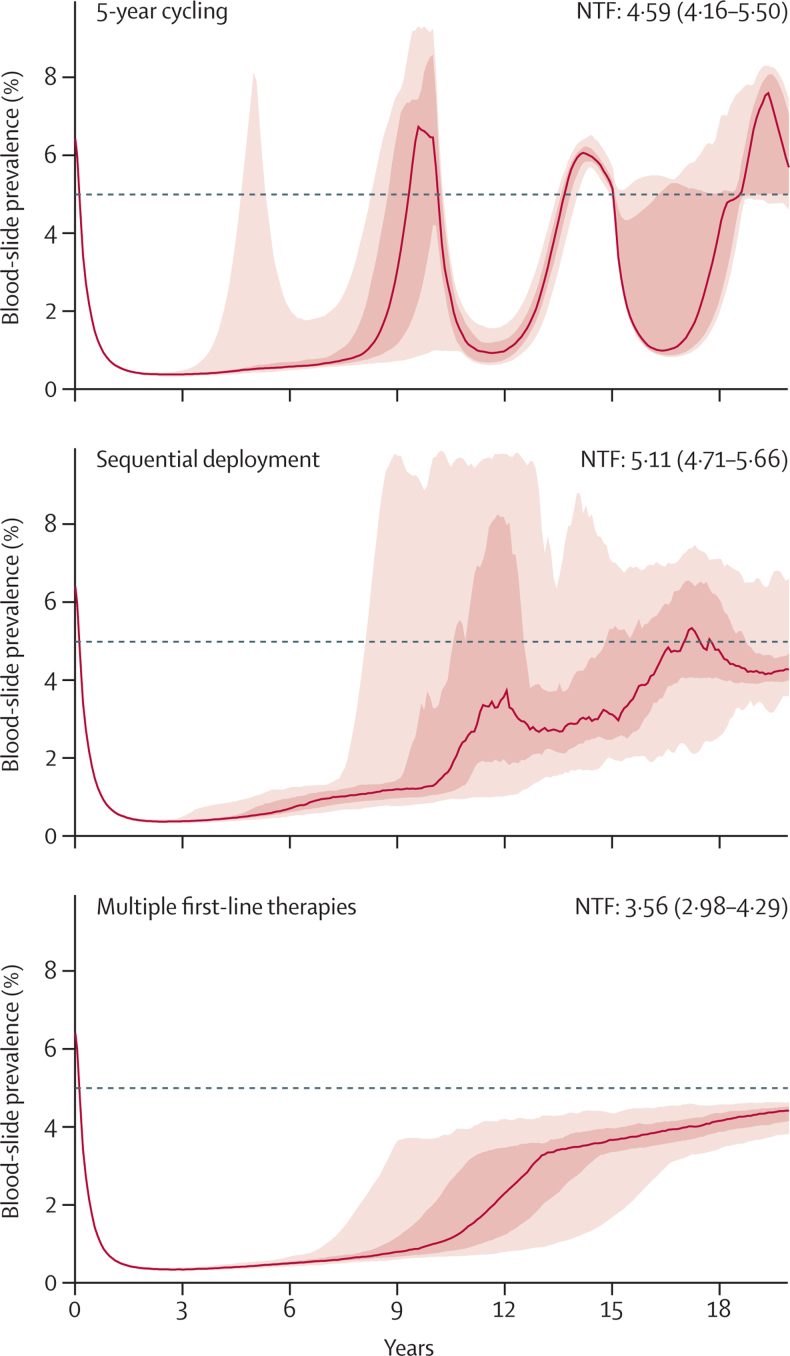
Expected patterns of drug-resistance evolution and corresponding malaria prevalence for three treatment regimens We ran 100 stochastic simulations in a population of 1 million individuals, in a low-transmission setting (entomological inoculation rate=1·3) with 60% treatment coverage and an assumed cost of resistance of 0·5% for resistant genotypes. The mutation rate is assumed to be highest for intermediate drug concentrations. The red line shows the median prevalence across 100 simulations, and the red regions show the IQR and 90% range. The median number of treatment failures (NTF) with IQRs are shown in each panel (p<0·0001 when comparing multiple first-line therapies to the other two strategies). The dashed line shows 5% prevalence. For the cycling and sequential strategies, after emergence of a novel drug-resistant type, an epidemiological rebound sometimes causes prevalence to reach higher-than-expected levels (here, >6%) for short periods.

Use of MFT had consistently better population-level outcomes than cycling and sequential deployment strategies, and the superior performance of MFT was robust in various transmission, clinical, and evolutionary settings. As noted in previous modelling studies,^18^, ^19^ an MFT strategy creates a more variable environment than drug cycling, which results in delayed resistance emergence and slower drug-resistance evolution. This effect is seen even when allowing for free recombination among resistance loci, which was previously raised as a potential concern when assessing the benefits and drawbacks of MFT strategies.^28^ Figure 1 shows typical evolutionary and epidemiological trajectories for malaria in the three treatment strategies we assessed.

**Figure 2:**
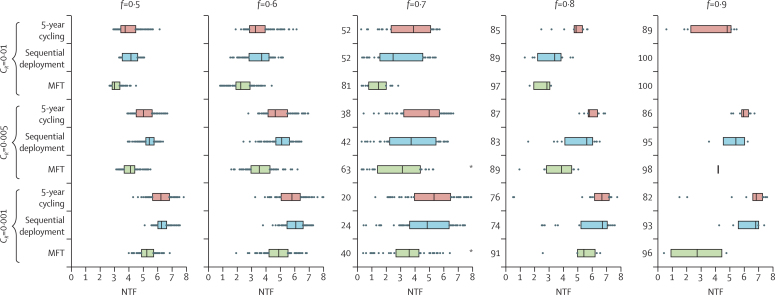
Median number of treatment failures (NTFs) for different strategies, different costs of resistance (*C*_*R*_), and different treatment coverages (*f*) Each row shows the NTF results of 100 model simulations with bars spanning the IQR. NTF values are lower for multiple first-line therapies than for cycling or sequential strategies; all p<0·0001, except for the comparisons *, for which p=0·01. For *f*≥0·7, the NTF distributions have a bimodal shape, with NTF<0·5 corresponding to simulations that achieved extinction or near-extinction; the numbers on the left-hand side of each boxplot show the counts of these extinctions or near-extinctions, and the IQRs are plotted only for simulations that did not result in extinction. Simulations assume that three artemisinin combination therapies with 95% efficacy are used in a low-transmission setting with an entomological inoculation rate of 1·3. Drug resistance mutations have their highest probability of fixation at intermediate drug concentrations.

**Figure 3:**
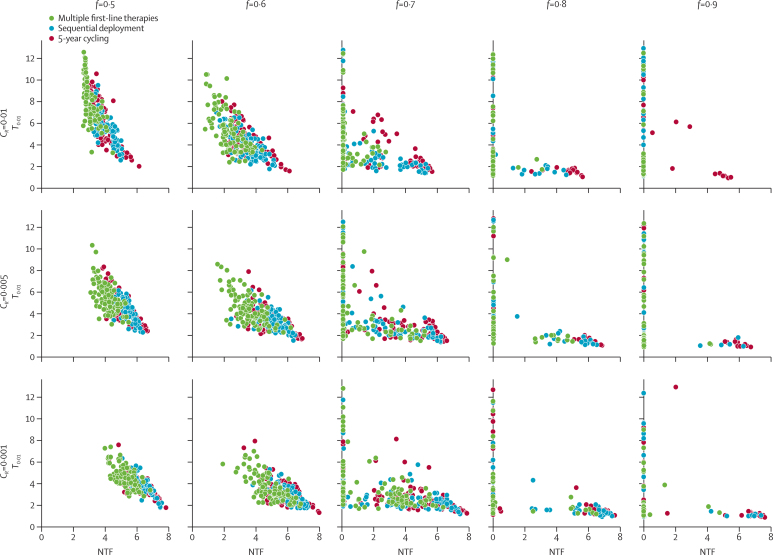
Number of treatment failures (NTF) plotted against the time taken to reach an average of 1% resistance frequency in the population Variance in time-to-emergence is greater for MFT strategies than for cycling or sequential strategies, resulting in a subset of simulations with long emergence times.

A strategy comparison in a low-transmission setting corresponding to an entomological inoculation rate of 1·3 infectious bites per person per year shows significant variation, owing to the stochastic nature of the model, in the number of treatment failures (figure 2) and the time until resistance emergence (*T*_0·01_; figure 3). In some high-coverage settings, treatment drives the parasite population to extinction before drug resistance emerges. When resistance evolves (0·5≥*f*≥0·7), MFT strategies have median numbers of treatment failures 16–41% lower (p=0·003; Mood's test) than either the sequential strategy or the cycling strategy; this difference is larger for higher costs of resistance. Additionally, elimination was seen more often with MFT strategies since these strategies preserve full drug efficacy for longer than do the cycling or sequential strategies. Figure 3 shows that low numbers of treatment failures are closely associated with long times to resistance emergence. MFT strategies have a higher mean and more variation in their associated time-to-emergence (an expected outcome for a waiting process) than do cycling strategies, resulting in a higher proportion of simulations in which resistance emerges very late or not at all during the 20-year model simulation.

**Figure 4:**
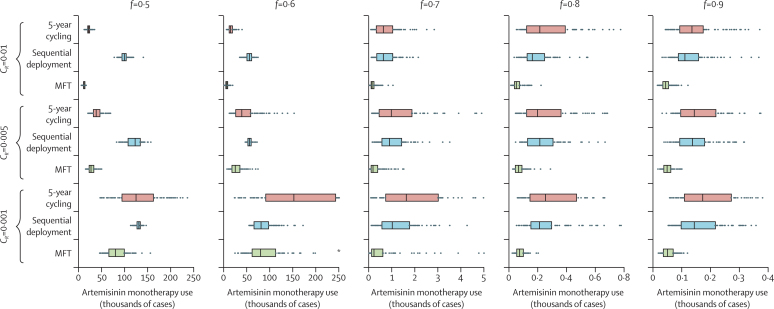
Comparisons of artemisinin monotherapy use for multiple first-line therapies (MFT), 5-year cycling, and sequential deployment Artemisinin monotherapy use values are shown for different costs of resistance (*c*_*R*_), and different treatment coverages (*f*). Each row shows the artemisinin monotherapy results of 100 model simulations, with bars spanning the IQR. Artemisinin monotherapy use values are lower for MFT (all p=0·001) except for the comparisons corresponding to *. Simulations assume that three artemisinin combination therapies with 95% efficacy are used in a low-transmission setting (entomological inoculation rate=1·3). Partner-drug resistance mutations have their highest probability of fixation at intermediate drug concentrations. Artemisinin monotherapy use decreases with treatment coverage because prevalence is lower when more individuals are treated.

Because these model simulations rely partly on prediction of rare events (emergence of artemisinin resistance), we ran a separate set of simulations to quantify risk of artemisinin resistance using the artemisinin monotherapy use criterion: the total number of cases during the 20-year simulation in which a partner-drug-resistant infection was treated with an ACT (figure 4). Excluding one comparison set in this figure, the median artemisinin monotherapy use values for MFT strategies were 28–78% lower (p<0·0001; Mood's test) than for cycling or sequential use, suggesting that MFT strategies might have a much lower risk of selecting for de-novo artemisinin resistance. MFT strategies delay and decelerate partner-drug resistance evolution, thus prolonging the time that artemisinin-based drugs are used as combination therapies with both components effective.

We did a comprehensive set of simulations to vary transmission setting, biting rate heterogeneity, treatment coverage, relation between drug concentration and mutation, drug half-life, and fitness cost of resistance. Excluding extinctions, MFT strategies were associated with the lowest number of treatment failures of any treatment strategy in 4434 (86·2%) of 5146 simulations; extinctions were most common with MFT. The median reduction in treatment failures achieved by MFT was 10·6% (IQR 2·9–20·0; p<0·0001) compared with a 5-year cycling strategy, and 9·6% (3·0–18·6; p<0·0001) compared with sequential deployment (appendix).

**Figure 5:**
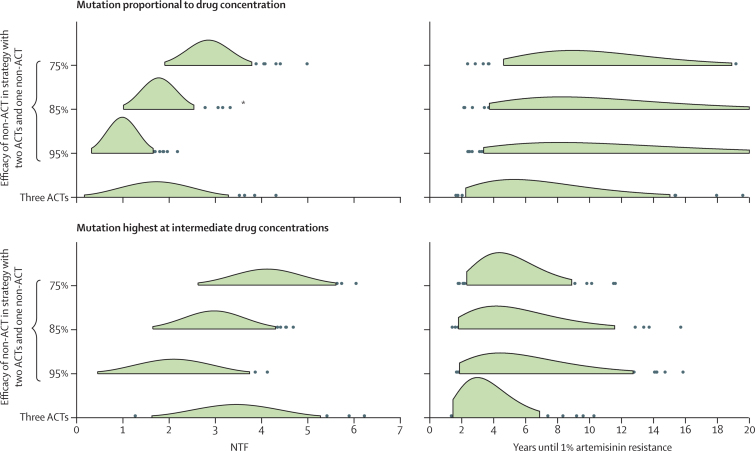
Comparison with a non-artemisinin combination therapy (ACT) drug included in a multiple first-line therapies (MFT) strategy Comparisons between MFT strategies with three ACT components and MFT strategies in which one of the components in the treatment strategy is not an artemisinin-based therapy. Results of 100 simulations for each strategy are summarised as normal distributions for the number of treatment failures (NTF; shown in green out to ±2σ), and as gamma distributions for the time until frequency of artemisinin resistance reaches 1% (central 90% of distribution shown in green). Distributions of NTF and the time until 1% artemisinin resistance for three ACTs are significantly different (p=0·01) from the comparator distributions in which one non-ACT is used, except for the comparison labelled *, for which there was no difference. The upper panels use a mutation model in which mutation rate is proportional to drug concentration. The lower panels show simulation results when the mutation rate increases at intermediate concentrations. Simulations assume that three ACTs with 95% efficacy are used in a low-transmission setting (entomological inoculation rate=1·3). In the simulations with two ACTs, the shorter half-life ACTs were used (minimising selection pressure), and the non-ACT therapy is assumed to be a combination therapy with components that have 7-day and 10-day half-lives. Treatment coverage is *f*=0·6.

In addition to deploying MFT, we considered other possibilities that would enable us to preserve the efficacy of artemisinin-based drugs for as long as possible. From the analyses presented here and basic evolutionary theory, alleviating parasites from artemisinin drug pressure should slow down evolution of artemisinin resistance. Use of a non-ACT alongside two artemisinin-based therapies in an MFT strategy should have this effect. Clearly, the major consideration for such a strategy is whether the non-ACT component is as effective and safe as an ACT. If this individual-level acceptability criterion is met, an MFT strategy with a single non-ACT therapy^29^ could substantially extend the lifespan of artemisinins. Figure 5 shows the lower number of treatment failures and longer useful therapeutic life that would be associated with this strategy, the lower number of treatment failures being a direct result of a longer time to emergence of artemisinin resistance. In these simulations, treatment coverage was 60%; thus, even in the worst-case scenario of 75% efficacy for a non-ACT therapy, only 4% of symptomatic malaria cases in the model had treatment failure as a result of not receiving an ACT. In the six scenarios in figure 5, the median time to emergence for a strategy with a non-ACT was 43–72% longer (p=0·001; Mood's test) than in a strategy with three ACTs. Additionally, with a mix of two ACTs and one non-ACT, a third ACT would be preserved in case one of the partner drugs began to fail early.

We investigated the possibility of targeting different age groups with different ACTs and changing usage frequencies for ACTs according to partner-drug half-life. We identified no simple method to optimise either of these strategies. A specific challenge with an age-based treatment strategy is the changing age profile of symptomatic infections as transmission intensity decreases over time. Results of previous studies^19^, ^30^, ^31^ have described selection pressure for drug resistance increasing with lower transmission. However, our age-structured model shows that this effect has a strong interaction with age, with younger age groups exerting less selection pressure, and older age groups exerting more selection pressure, as transmission decreases (appendix). Adjustment of the distribution strategy according to half-life did not seem to have a large effect on long-term treatment outcomes (appendix).

## Discussion

Our analysis suggests that use of MFT strategies should result in improved population-level treatment outcomes, delayed resistance emergence, and slowed resistance evolution, as seen in previous, more general analyses.^18^, ^19^, ^20^, ^22^ Additionally, the major prevailing concern about MFT strategies—that they would enable recombination to generate multidrug-resistant types earlier than other strategies—proved not to be true in any of our simulations. The *P falciparum* transmission model we developed and validated for this study provides outcomes in accordance with expectations based on evolutionary theory: that challenging the parasite with an environment presenting several simultaneous lethal challenges significantly increases the time the parasite needs to defeat all of them.

Since population-level treatment strategies such as the ones assessed here will never be testable in the field, model-based recommendations might be the only evidence available to plan optimum distribution of antimalarial therapies. If the median predicted benefits are a 16–41% reduction in treatment failures during a 20-year period and a more than 40% increase in the useful therapeutic life of ACTs, model-based recommendations could be considered sufficient evidence for management of distribution and usage patterns of antimalarial drugs. As elimination campaigns move forward, MFT strategies might enable elimination efforts to succeed before a fully artemisinin-resistant genotype emerges.

The key principle in the strategy comparisons presented here is conservation of drug efficacy.^32^ As drugs are used more sparingly, drug efficacy is prolonged, and an MFT strategy enables individual drugs to be used sparingly without reducing the total number of patients intended to treat. The scenario presented in figure 5 specifically considers the conservation of artemisinin efficacy, and the potential for extending the useful therapeutic life of artemisinin drugs by pairing them with other highly efficacious drugs in an MFT strategy. This conservation approach is logical from an evolutionary perspective, but ethical implications will need to be assessed carefully since some patients could be treated with a therapy with non-optimum measured efficacy. To improve the chances that such a strategy meets the highest medical and ethical acceptability criteria, the risks for patients treated with a non-ACT would need to be mitigated, possibly through frequent follow-up and availability of second-line treatments.

As in all past analyses of the dynamics of drug-resistant parasites, measuring fitness costs^33^, ^34^, ^35^, ^36^, ^37^, ^38^ of drug-resistant genotypes is crucial for prediction of the spread of resistance. For artemisinin resistance, the dynamic picture (2002–13) of the spread of *kelch13* resistance-associated alleles in Cambodia is the best starting point to investigate fitness costs. However, the fitness cost of any future hypothetical resistant genotype is impossible to predict. To be conservative, fitness costs in our analyses were varied between 0·1% and 1·0%, because scenarios with much higher fitness costs would result in a bigger advantage of MFT compared with cycling or sequential strategies.

In-depth critiques of model structure and validations are necessary when interpreting results from mathematical models. The model presented here does not take into account fine-scale spatial structure, mosquito dynamics, gametocyte dynamics, genotype-specific drug efficacies, or seasonal or climate effects, but all these features should clearly be considered when planning treatment campaigns and elimination strategies. Continual development and validation of models by adding in realistic features that are known to have important effects on malaria transmission and evolution are crucial so that every iteration of development brings the model closer to resembling the real-world epidemiology of malaria. Comparison of these results with those of other models (appendix) is crucial to test robustness. Analyses in many types of transmission settings need to be done to ensure that specific policy recommendations are optimum. Our results suggest that MFT's relative benefits are smaller in higher transmission settings (appendix), but absolute benefits do not show this pattern consistently.

Potential caveats about the benefits of MFT strategies need to be addressed and evaluated. First, strategy comparisons will probably be sensitive to the parity of available therapies. In a scenario in which one ACT has much higher efficacy than the other two, use of the high-efficacy therapy first might be prudent since this strategy would lower the parasite population size more quickly (across all hosts) than use of a lower efficacy therapy, and would lower the probability of random mutation generating a drug-resistant genotype. Recommended and available ACTs have similar efficacies,^39^ but this is location-dependent.^40^ Scenarios in which, for example, two treatments with 90% and 98% efficacy have different predicted effects on a desired epidemiological outcome such as elimination will need to be assessed on a case by case basis. Second, in choosing the optimum strategy we need to consider the multitude of resistance effects caused by individual loci, an important example being position 76 in the chloroquine resistance transporter gene (*pfcrt*), in which the wild-type allele (K) confers lumefantrine resistance and the mutant allele (T) confers amodiaquine resistance.^41^, ^42^ Cycling strategies usually have worse evolutionary outcomes than do other strategies because they drive evolution of individual resistance types more quickly, but in the case of K76T, amodiaquine resistance driving lumefantrine sensitivity might mitigate this problem. These effects might be present for other loci.^43^, ^44^

Implementation, compliance, and operations will be the next important areas of focus if MFT strategies are accepted as the best strategy for distribution and prescription of antimalarial drugs. Operationally, MFT would have several advantages because it removes the need for large system-wide changes in drug policy and avoids problems of obsolete drug stocks. Whether randomly assigning therapies should be done by location, day of week, a true randomisation scheme, or another method is unknown. Variation in drug purchase patterns from private and public sectors^45^, ^46^ will necessitate different implementations, and compliance monitoring will be challenging in contexts with high levels of private sector drug purchases. Nevertheless, some countries have successfully managed roll-out of multiple ACTs,^47^, ^48^, ^49^, ^50^ showing the feasibility of locally determined drug distribution and flexible treatment guidelines on the basis of changing epidemiology. A commitment to assessing the effectiveness of new population-level malaria treatment programmes and a willingness to adapt approaches will be crucial to maximise the benefits of MFT to global malaria policy.
