# Supplementary material for: Optimum population-level use of artemisinin combination therapies: a modelling study
Source: Lancet Glob Health. 2015 Nov 4;3(12):e758–66. doi: 10.1016/S2214-109X(15)00162-X (PMC4641185; doi:10.1016/S2214-109X(15)00162-X)
Supplement: Supplementary appendix [file mmc1.pdf]

THE LANCET

Supplementary appendix

This appendix formed part of the original submission and has been peer reviewed. We post it as supplied by the authors.

Supplement to: Nguyen TD, Olliaro P, Dondorp AM, et al. Optimum population-level use of artemisinin combination therapies: a modelling study. *Lancet* 2015; published online Nov 3. http://dx.doi.org/10.1016/S2214-109X(15)00162-X.

Appendix for "Optimal population-level deployment of artemisinin combination therapies"

TABLE OF CONTENTS

The two first sections of the Supplementary Appendix contain a description of the implementation of the individual-based model ("microsimulation"); this description includes details on the mechanisms and assumptions in the model that are related to transmission, clinical progression, immunology, pharamacokinetics and pharamacodynamics, evolution, and demography. The second section below shows how population-level treatment strategies were compared.

1 Model Description . . . 2
2 Strategy Comparison and Evaluation Criteria . . . 4

Sections 3 through 9 contain descriptions of model parameters where model inputs can be directly obtained from field or clinical data.

3 Gametocytaemia and Infectivity . . . 6
4 Duration of Infection . . . 7
5 Probability that an Infectious Bite Causes an Infection . . . 7
6 Age-specific All-cause Mortality and Malaria Mortality . . . 8
7 Age Structure . . . 10
8 Parasite Density Levels . . . 10
9 Pharmacokinetics and Pharmacodynamics . . . 11

Sections 10 through 16 describe model validations for behaviors that cannot be input directly into the model. For example, clinical episodes decrease with age more quickly in high-transmission settings than in low-transmission settings, but the model cannot be forced to exhibit this behavior; this behavior is a consequence of the interaction between population-level immunity and entomological inoculation rate (EIR). In the sections below we validate that these qualitative features of malaria epidemiology are observed in our model.

10 Model of Immune Acquisition and Symptoms . . . 13
11 Incidence of Clinical Episodes by Age . . . 15
12 Prevalence of Symptoms and Blood-Slide Prevalence (φ value) . . . 19
13 Relationship between EIR and Malaria Prevalence . . . 20
14 Prevalence by Age . . . 22
15 Multiplicity of Infection . . . 23
16 Biting-Rate Heterogeneity and Prevalence . . . 25

Section 17 shows the results of a general sensitivity analysis across several key epidemiological and pharmacological parameters in the model. Section 18 presents several additional strategy comparisons and exploratory analyses of model robustness. Section 19 compares the results presented in this paper to those of Antao and Hastings (*Malaria Journal*, 2012).

17 General Sensitivity Analysis . . . 27
18 Additional Figures for Specific Sensitivity Analyses . . . 31
19 Comparison to Results in Antao-Hastings (2012) . . . 37

1 Model Description

The model is an individual-based stochastic microsimulation developed in C++ using a daily time-step and asynchronous updating. Individuals in the model carry certain basic attributes such as age, attractiveness to mosquitoes, presence/absence of malaria symptoms, level of parasitaemia (possibly none), the number of independent clonal parasite populations circulating in their blood, the presence/absence of parasites in the liver (before a blood-stage infection), level of acquired immunity to malaria, and drug levels in the blood. These are described in more detail below. The population size in the model is set to one million, as larger population sizes showed no difference in qualitative dynamics. The sensitivity analyses in section 17 were carried out with populations of 500,000 individuals (for quicker simulation runs). Typical simulations for our present analysis were run to equilibrium (initially checked visually, but all simulations reached this equilibrium within 4000 days) with no treatment, after which treatment was initiated – for a fraction f of symptomatic malaria cases (known as the "treatment coverage") – and the simulations were run for another twenty years. The treatment coverage parameter f was varied between 0.5 and 0.9 in the simulations. Asymptomatic cases were not treated in our simulations. Our microsimulation is meant to model the dynamics of *Plasmodium falciparum*.

Blood-stage Parasitaemia. Each host i is associated with an asexual parasite density D_i, which is the sum of the densities of the individual clonal parasite populations in that host. If there are c_i clonal parasite populations inside host i, then we let $j = 1,\ldots, c_i$ index these clonal populations. We let R_j be the resistance profile of clonal population j, where R_j is the set of drugs to which the corresponding parasite population carries resistance (using the index-set notation of Andreasen et al [1]). As an example, $R_3 = \emptyset$ indicates that the third clonal population in this host does not carry any drug resistance genes, and $R_4 = \{1, 2\}$ indicates that the fourth clonal population in this host carries resistance to drugs 1 and 2. The parasite density inside host i is then described by

$$D_i = \sum_{j=1}^{c_i} w_{R_j}\, \delta_{j,i}\, \gamma_{j,i}$$

(1)

where w_{R_j} is the relative fitness of a resistant parasite with resistance profile R_j; the fitness of a sensitive parasite is defined as $w_\emptyset=1$. The quantity $\delta_{j,i}$ is the parasite density of clone j in host i, and $\gamma_{j,i}$ is the relative gametocyte production of clone j, with one corresponding to normal production and zero to no production. Typically, $\gamma_{j,i}$ is set to zero for the first four (children) or six (adults) days of an infection, and then fixed at one after gametocytes begin to be produced; this is slightly earlier than true gametocyte production, the reason being that we do not model the tail-end of a cured infection when asexual parasitaemia is zero but gametocytaemia stays positive for one week or more [2]. Essentially, the model shifts the gametocytaemic period to be several days earlier so that infected hosts have the same number of transmissible days as they would for real infections. This adjustment does not affect our simulations as we do not model gametocytocidal drugs such as primaquine. The force of infection of resistant type R at time t is

$$\Lambda_{t,R} = \beta \sum_{\text{all hosts } i} g(D_i) \cdot b_i \cdot \left(\frac{w_R\ \sum_{j:R_j=R} \delta_{j,i}\, \gamma_{j,i}}{D_i}\right)$$

(2)

where b_i is the biting attractiveness of host i (drawn from a gamma distribution with coefficient of variation equal to 2.0 [3]), the function g describes the saturation of transmission probability with increasing parasite density [4] (see section 3), and β is a scaling factor that we vary to obtain a particular entomological inoculation rate (EIR) in a given simulation. Recombination can occur when a mosquito bites a host with multiple clonal infections. We assume that interrupted feeding on multiple hosts does not occur; the frequency of interrupted feeding by non-infected mosquitoes has been estimated to be as high as 10% [5–7] but our previous modeling analysis [8] indicated a weak relationship between

outcrossing rate and the evaluation criteria we measure here (NTF and $T_{.01}$; see section 2). It is assumed that drug resistance genes are located on different chromosomes. The parasite densities in the above equation already take into account a single generation of segregation and recombination that occurs inside the mosquito.

Parasitaemia levels are set to 20,000 parasites per microliter at the beginning of a symptomatic episode of malaria. Asymptomatic cases have their parasitaemia levels initialized at 1000 parasites per microliter, after which the parasite population size is slowly reduced by the immune system; the minimum and maximum durations of asymptomatic parasitaemia observed in the model are 60 days and 281 days [9–11], and this varies with the host's immune status. For a multi-clonal infection presenting with clinical malaria, symptoms are caused by one clonal parasite population (typically, the most recent bite) reaching a density of 20,000 parasites per microliter, while the other parasite population sizes stay at levels of <1000 parasites per microliter.

Transmission model. Genotype-specific forces of infection are stored in the simulation for 11 days, and new infections are generated by drawing a Poisson number of hosts based on each genotype's force of infection 11 days ago (this 11-day lag mimics the course of parasite development in the mosquito [12–14]). Once this number has been drawn, the specific hosts to be infected are chosen with replacement relative to their biting weights b_i, and seven days later, after growth and development of liver-stage parasites, these hosts will have a newly emerged blood-stage parasite population (50,000 total parasites).

Immunity Model. We implement an immunity model in each host that describes the general level of acquired immunity that a host has to *P. falciparum* malaria; specific immunity is not modeled. This model's immunity variable M ranges from zero to one, and it is meant to be interpreted on a relative scale. Its two biological effects are that a higher level of immunity increases parasite clearance rate and decreases the chances that a new infectious bite leads to a clinical episode of malaria, similar to Filipe et al [15]. The immunity-dependent parasite clearance rate is

$$D_{t+K} = w_R[0.8036 \cdot (1 - M_t) + 0.9572 \cdot M_t]^K \cdot D_t$$

where D_t is the parasite density at time t, and w_R is the fitness of the given strain (1 for the drug-sensitive strain, 1 – c_R for a single-drug resistant strain; see drug-resistance description below). The parasite density is typically updated asynchronously every seven days (K=7) in the simulation; validations were done to ensure this is not very different from daily updating. If an individual has another event occurring (e.g. new infection, clearance of a different parasite clone) then the parasite density is updated earlier than the scheduled 7-day interval. The parameters in this equation are calibrated so that infections are a maximum of 281 days and minimum of 60 days. Details of this calibration are presented in Section 4. The effect of immunity of symptoms development is described in Sections 10 and 11 of this document.

Immunity is acquired, at an age-specific rate, when a host is parasitaemic, and immunity wanes at an age-independent rate when a host is uninfected. Immune acquisition occurs slowly for children under ten, and it is calibrated so that chronically infected children achieve high levels of immunity by age nine or ten. Naïve adults acquire high-levels of malaria immunity after 2.5 to 3.0 years of chronic infection. For uninfected individuals, substantial immune loss occurs after 1.5 to 2.0 years. Sections 10 to 12 contain the equations for immune loss and immune acquisition, as well as a descriptions/validations of how parameters were chosen.

Clinical/Symptomatic Malaria. When an individual in the model acquires a new malaria infection, that host will progress to clinical or symptomatic malaria with probability P_{clin}. This is modeled as

$$P_{clin} = \frac{0.99}{1 + (M/0.4)^z}$$

where M is the immune level. This clinical feature of the model was calibrated together with the immune-acquisition component of the model so that age-specific clinical incidence in children matched field observations at different transmission intensities [16–20], and the parameter in the exponent was set to z = 4.0 (see sections 10 to 12).

Pharmacokinetics and pharmacodynamics (PK/PD). Drug action was modeled with a standard concentration-effect curve, and drug clearance was modeled with a standard exponential decay (section 9). Daily killing rates, or parasite reduction ratios, were obtained from past PK/PD studies [21–30]. Variable drug absorption was included so that different individuals had different starting concentrations of drug (this allowed treatment to fail sometimes); the coefficient of variation of the starting drug concentration was varied between 0.1 to 0.4. This coefficient of variation and the slope of the concentration-effect curve were varied for each specific therapy, to obtain a desired efficacy at 28 days. As an example, the maximum daily killing rates for artemether, artesunate, and dihydroartemisinin were set to 99.9% per day. For artemisinin combination therapies, the standard deviation for the initial concentration of both drug components was set to 0.4, resulting in an efficacy of 95%, and these parameters were used for ACTs in the simulations presented here.

Drug resistance evolution. Evolution in clonal parasite populations is modeled by allowing a parasite population to mutate from sensitive to resistant to drug x, only in the presence of drug x; as in previous modeling studies, this model behavior really represents the fixation of a new mutant, but we keep the terminology of a "mutation rate" to be consistent with past modeling literature. In the simulation, mutation occurs on a daily basis with a certain daily probability. For the majority of drugs, these probabilities are unknown. Hence, the probabilities in the model are set so that resistance evolves, under high drug coverage, during the 20-year time span that the model is run. These mutation parameters were set to be the same for all resistant genotypes, including those encoding resistance to the partner drugs, as the relative magnitudes of these mutation rates are unknown. In the model version presented in this paper, drug-resistant genotypes are completely resistant to the action of the drugs they are resistant to.

Mutation rates from drug-sensitive to drug-resistant genotypes are a function of the drug concentration. When the drug concentration is zero, the mutation rate is set to zero. When the drug concentration is a full dose, the mutation probability is μ. We introduce a parameter k to determine whether the probability of drug resistance emerging and fixing is higher or lower at intermediate drug concentrations. Under a simple linear model, the mutation probability falls linearly with drug concentration (k=0.5) so that the mutation rate is $\mu/2$ at half concentration. We also test a model where the mutation probability is twice as high at half-concentration than at full concentration (k=2), and another where the mutation probability is four times as high at half-concentration (k=4). The scenarios in Figures 1 to 3 in the main text, and the bottom panels of Figure 4 were simulated with k = 4.

Each resistant genotype is assigned a fitness cost c_R (that varies between 0.1% and 1.0%), and for multiple-resistant genotypes fitnesses are multiplicative. The fitness component in the force of infection equation (2) is defined as $w_R = 1 - c_R$.

2 Strategy Comparison and Evaluation Criteria

Assuming that three ACTs with different partner drugs are available for treatment, three population-level treatment strategies were compared in the simulations. The half-lives and efficacies of the therapies in the simulation were set to mimic the characteristics of artesunate-amodiaquine (ASAQ), artemether-lumefantrine (AL), and dihydroartemisinin-piperaquine (DHA-PPQ).

Multiple first-line therapies (MFT). In a strategy of multiple first-line therapies, one-third of individuals are treated with AL, one third with ASAQ, and the final third with DHA-PPQ. If the treatment coverage parameter is f = 0.6, this means that 40% of individuals would not be treated for a symptomatic malaria infection (due to lack of access, inability to pay, not reporting to a clinic or pharamacy, etc.). The

model would then have 20% of symptomatic individuals treated with the first ACT, 20% with the second ACT, 20% with the third ACT, and 40% would receive no treatment. Individuals are not pre-assigned to these groups; the `random draw' is random for every treatment and for every individual. In other words, the same individual can receive three different ACTs for three different symptomatic malaria episodes. One of the reasons that a "pre-assignment MFT policy" was not considered was that we believe this type of implementation would be operationally more difficult than simply distributing therapies randomly. In rural malaria-endemic settings, individuals are unlikely to know their medical history, carry their ID card, or even accurately know an identifying piece of information such as their birthday.

Five-year cycling. In a 5-year cycling strategy, a single ACT is used in the population at any one time and the ACTs are rotated out and switched every five years. We compared several cycling strategies in our previous publication [8] (2-year cycling, 3-year cycling, etc). Here, we choose five years as a feasible schedule which could be implemented at a national level in an endemic country. Shorter cycling periods are of course associated with improved outcomes, as they create more variability for the parasite population and slow down the pace of resistance evolution. The shorter the cycling period, the closer this strategy approximates an MFT strategy.

Sequential Deployment. The third strategy is meant to mimic the status quo approach of rotating therapies out when they begin to fail. We call this 'sequential deployment', and under this strategy switches occur when the treatment failure rate (using a 60-day moving average) reaches 10%, the WHO criterion for replacing first-line therapies [31]; a one-year delay is built in to this strategy as it is known that switching first-line drugs at the national level can take a significant amount of time and effort [32]. This is optimistic, but we used this one-year delay to compare the best possible sequential deployment strategy against the two alternatives. In our previous publication, this strategy was called 'adaptive cycling' [8].

Multiple first-line therapies with one non-ACT component. For the final figure in the paper we evaluated an MFT strategy with two ACTs (ASAQ and AL) and one non-ACT therapy. The non-ACT component in this strategy was a combination therapy whose components have 7-day and 10-day half-lives, mimicking the pharmacodynamics of chloroquine and sulfadoxine-pyremethamine.

Four evaluation criteria are used:

NTF. The number of treatment failures per 100 individuals per year over the 20-year course of the simulation. This measure is discounted with a 3% annual discounting rate. Non-treatments are counted as treatment failures; if this were not done, then policies with low coverage (f) would appear optimal as they would treat the fewest people and drive resistance evolution at the slowest rate.

AMU. Artemisinin monotherapy use is a risk measure describing how exposed to resistance evolution the artemisinins are under a particular strategy. "Exposed" in this context means "not protected by the partner drug". To compute the AMU measure, we run a separate set of simulations with the artemisinin-resistance mutation parameter set to zero, and we count all treated cases in which an individual with parasites resistant to partner-drug x is treated with an ACT containing the same partner drug x. We call this type of treatement *de facto* artemisinin monotherapy use because only the artemisinin component is acting in this situation. AMU is simply an absolute count of all cases of *de facto* artemisinin monotherapy use during a 20-year simulation.

We recorded three types of AMU measures in our simulation: by person, by parasite clone, and by person but weighted by the number of parasite clones. Consider an individual with 5 clonal *P. falciparum* infections in the blood, two of which carry amodiaquine resistance. If this person were treated with ASAQ, then we could count this as 1 AMU ('by person' measure), 2 AMU ('by parasite clone'), or 2/5 AMU (weighted measure). All three measures had similar behaviors when comparing treatment strategies. In Figure 3 of the main paper, we present the weighted measure.

We do not discount the AMU measure as we are trying to compare the relative probabilities across treatment strategies that, over a 20-year period, a novel artemisinin-resistance genotype could emerge and establish itself. Alternatively, AMU can be thought of as the total amount of "selection pressure via artemisinin monotherapy" present during a 20-year period. Comparisons using a discounted AMU measure were qualitatively identical to those shown in Figure 3 of the main text.

UTL. In an MFT strategy, this is the time until the treatment failure rate reaches 10%. Treatment failure is computed in all simulations as a moving average over the past 60 days as this is what is likely to be picked up by surveillance systems. For the cycling and sequential strategies, the UTL is defined as the total time during the 20-year simulation that the treatment failure rate is below 10%. Note that the treatment failure rate computed in the simulation includes the usual expected treatment failure even when the parasite population is drug-sensitive. For this reason, the UTL would not be a useful measure for a drug or therapy with 91% efficacy as 10% treatment failure could be reached very quickly.

$\boldsymbol{T_{.01}}$. The time at which the "genotype frequency of all resistant alleles" reaches 1% in the population. If four drugs are being used in the population, then each parasite can be a single-resistant, a double-resistant, a triple-resistant or a quadruple-resistant. If p_1 is the frequency of single-resistant genotypes, p_2 the frequency of double-resistant genotypes, etc., then the "genotype frequency of all resistant alleles" is

$$\frac{1}{4}p_1 + \frac{1}{2}p_2 + \frac{3}{4}p_3 + p_4.$$

The frequencies are calculated across parasite clones. This quantity is equivalent to the probability that if a parasite genotype is chosen at random and a drug is chosen at random, that the parasite will carry resistance to that drug.

Statistics. When two strategies were compared, typically 50 or 100 model simulations were run under each strategy. To compare the distribution of 100 NTF values from one strategy to 100 NTF values from another strategy, four statistics were used. The 25th, 50th, and 75th quantiles of the distributions were compared, by assuming that the two distributions had the same quantiles (null hypothesis), counting NTF values that fell above/below the common quantile for both strategies, and comparing the resulting four numbers with a χ^2 test with one degree of freedom (Mood's test). In addition, the Mann-Whitney test was used to compare the ranks of the two distributions. When we write $p < 10^{-6}$ for a particular strategy comparison, this indicates that all four p-values were smaller than 10^{-6}. When we write $p > 0.10$ for a particular strategy comparison, this indicates that all four p-values were larger than 0.10.

3 Gametocytaemia and Infectivity

Ross and colleagues developed a statistical model that described the relationship between host infectivity to mosquitoes and asexual parasite density and fit that model with data from 392 neurosyphilis patients treated with malaria therapy [4]. In their model, the relationship between gametocyte density and asexual parasite density was described as

$$\ln(y_g) \sim Normal(\ln(\rho\Upsilon), \sigma_g)$$

where y_g is the density of functional female gametocytes in an individual, Υ (upsilon) is the asexual parasite density, ρ is the parameter that tells us the proportion of asexual parasites that have developed into gametocytes, and σ_g is the standard deviation. Then the probability that at least one functional gametocyte is taken up is defined as

$$\Pr\left(y_g > y_g^*\right) = \Phi\left[\frac{\ln(\rho\Upsilon) - \ln\left(y_g^*\right)}{\sigma_g}\right] = \Phi\left[\frac{\ln(\Upsilon)}{\sigma_g} + \rho^*\right]$$

where y_g^* is the gametocyte density (per microliter) required for a mosquito to take up at least one functional gametocyte during one blood meal; Φ is cumulative distribution function (CDF) of the standard normal distribution and $\rho^* = \frac{\ln(\rho) - \ln\left(y_g^*\right)}{\sigma_g}$. If the volume of the blood meal is assumed to be 3μl [33] then $y_g^* = \frac{\rho}{3}$. And, if the sex ratio between male and female gametocytes is 1:1 then the probability that a mosquito is infected by taking up both male and female gametocytes is $\Pr\left(y_g > y_g^*\right)^2$. The best fit values from this data set were $\rho = 0.00031$ (95% CI: $0.00027 - 0.0036$) and $\sigma_g = 3.91$ (95% CI: $3.72 - 4.10$).

In our simulation, we use this Ross et al (2006) function $\Pr\left(y_g > y_g^*\right)^2 = \left(\Phi\left[\frac{\ln(\Upsilon)}{3.91} - 1.7852\right]\right)^2$ to describe the per-bite infectivity of an individual with an asexual parasite density Υ. An individual with a parasite density of 10/μl will have approximately a 1% chance of infecting a mosquito. An individual with a parasite density of 1000/μl will have approximately a 25% chance of infecting a mosquito.

4 Duration of Infection

Using the malaria therapy data, Maire et al [10] computed the duration of an untreated infection. Of patients whose infection duration was longer than two months, the log-mean of the untreated infection durations for 47 patients was 5.13 (σ=0.80), which corresponds to a mean of 169 days (± 1σ range: 76 – 376). Eyles and Young [9] described the course of malaria infection in 22 neurosyphilitic patients with malaria therapy and presented an average duration of infection of 222 ± 117 days for patients where the entire course of continuous and intermittent parasitaemia was followed until they were defined as cured or cleared (6 months of zero parasitaemia). For a larger group of 38 patients, the initial course of continuous parasitaemia was calculated as 121 ± 58 days. These patients were normally untreated and occasionally received quinine treatment during clinical episodes.

In the World Health Organization's Garki Project Report [11], the daily clearance rate ranged from 0.002 to 0.018 (approximately, according to the Figure 31 of the report), corresponding to an infection duration between 55 and 500 days (in the absence of interventions). This graph also shows an increasing clearance rate with age which is likely due to the development of immunity in older individuals.

In our simulation, we define the duration of infection as the number of days that an untreated or inadequately treated individual clears all blood-stage parasites (by action of the immune system only). Using the reference data above, we set 60 days and 300 days as the lower and upper limits for infection duration, for fully immune and immunologically naïve individuals, respectively (see section 10 for the immunity model in the simulation). Because immunologically naïve individuals acquire some level of immune protection during the course of a malaria infection, a 300-day parasitaemia is never observed. The maximum observed parasitaemia in the simulation is 281 days for a naïve 1-year-old child and 197 days for a naïve 20-year-old individual.

5 Probability that an Infectious Bite Causes an Infection

From February 1986 to October 1987, Beire et al conducted an epidemiological and entomological investigation of malaria incidence in 809 children in Saradidi (western Kenya) to investigate the relationship between *P. falciparum* incidence and EIR [34]. Using clinical incidence reports and EIR measurements from household vector studies, the authors found that 7.5% (1 in 13) of sporozoite inoculations produced new infections in children in Saradidi. The measured EIR during this period was 0.75 infective bites per person per night or 273 bites per person per year.

A parallel study in Saradidi [35] indicated that adults were less susceptible to malaria compared to children given the same level of exposure. In the same village during a high-transmission period, 57 of 62 children (92%) experienced parasitaemia within 56 days and all children developed parasitaemia by day 84, while only 16% (day 56) and 58% (day 84) of adults developed parasitaemia. All participants received radical cure at the beginning of the study. Thus, the probability of developing parasitaemia after receiving an infectious bite likely depends on an individual's level of immunity (possibly sporozoite-blocking immunity, but this is not known).

In our simulation, we use a single parameter to define the probability that an infectious bite causes a new infection, as there are still not enough data to accurately measure the age dependence of this parameter. We keep this probability constant at 10% across all age groups.

6 Age-specific All-cause and Malaria Mortality

Considering DSS data from seven sites in sub-Saharan Africa from 2001-2005 [36], malaria mortality ranged from 2.5 to 8.2 deaths per 1000 children per year. Age-specific mortality rates are shown in Figure 1 of this paper. Extracting these numbers from the graphs and averaging across sites, we obtain the table below:

Age	All-cause Mortality	Malaria Mortality
0.1	170.2	27.4
0.3	58.7	23.8
0.6	60.6	28.3
0.9	62.4	31.5
1.1	52.3	23.4
1.4	43.7	19.6
1.6	37.8	16.2
1.9	31.4	13.4
2.6	21.3	19.2
3.5	10.1	9.6
4.4	5.0	4.2
5.4	4.9	2.7
6.5	3.8	1.9
7.5	3.0	1.2
8.5	2.7	1.8
9.4	2.9	1.7
10.4	2.2	1.0
11.5	2.1	1.5
12.5	2.2	1.6
13.4	2.3	0.9
14.4	1.8	0.8

Table S1. Averaged age-specific mortality (per 1000 person-years) from seven sites in Burkina Faso, Ghana, Kenya, Tanzania, and Mozambique. Note that because the ages are extracted from graph, they are not all exact integers.

Combining this with a longitudinal data set on 60,000 individuals in Burkina Faso from 1999 to 2003 [37], we summarize age-specific all-cause mortality and age-specific malaria mortality in the following table:

	<1 year	1-4 years	5-14 years	15-59 years	> 60 years	Reference
All-cause mortality	60.5	23.6	2.8	5.9	54.7	Becher et al
Malaria mortality	23.4	10.4	0.7	0.4	10.2	Becher et al
All-cause mortality	80.84	28.79	2.98	No data	No data	Abdullah et al
Malaria mortality	26.86	15.09	1.77	No data	No data	Abdullah et al

Table S2. Annual mortality (per 1000) by age group, data extracted from Becher et al [37] and Abdullah et al [36].

With these two datasets, we construct an age-specific mortality pattern (malaria attributable and non-malaria attributable) that we would expect to observe in a high-transmission region.

Age-Group	0-1	1-2	2-3	3-4	4-5	5-6	6-7	7-8	8-9	9-10	10-15	15-20	20-60	>60
All-cause Mortality	80.84	37.29	20.94	12.14	6.65	4.54	3.88	3.16	2.88	2.60	2.25	5.9	5.9	54.7
Malaria Mortality	26.86	18.36	14.07	11.02	5.51	2.93	1.92	1.63	1.59	1.53	1.27	0.4	0.4	10.20
Non-Malaria Mortality	53.98	18.94	6.87	1.12	1.14	1.61	1.95	1.53	1.30	1.07	0.98	5.5	5.5	44.5

Table S3. Expected age-specific mortality per 1000 persons per year in a high-transmission region.

The mortality not attributable to malaria is input directly into the demographic part of the simulation; however, malaria mortality depends on transmission and must be input as a per-case probability of death. In the simulation, we count failed treatments and non-treatments, but we do not distinguish between severe malaria and uncomplicated malaria in these cases. Using summary information from the WHO [38], we assume that the percentage of symptomatic malaria cases that progresses to severity is 5% for the 0-5 age group and 1% for individuals over five years of age.

In a meta-analysis of malaria clinical trials conducted through 2002, Myint et al summarized the mortality rates for uncomplicated and severe malaria, for which the probabilities of death were 0.03% and 13.9%, respectively [39]. These numbers are for treated patients. The assumptions in Goodman et al [38] have the under-five case fatality for severe malaria at 19.2% if treated and 50% if untreated; the over-five case fatalities used are 10% for treated patients and 25% for untreated patients. Depending on how many of the treated patients were receiving failing treatments and how many would progress to severe malaria under these circumstances, the malaria mortality for a "treated patient population" should be, approximately, between 0.1% and 1.0% of all malaria cases. For untreated patients, the total malaria mortality rate could be as high as 2.5% in under-fives and 0.5% in over-fives.

In our simulation we set the mortality rate for untreated malaria to 4% for age group 0-1, 2% for age-group 2-5, 0.4% for age-group 6-10, and 0.1% for older age groups. Successfully treated cases in our simulation result in zero mortality, and unsuccessfully treated cases have the same mortality profile as untreated cases. When evaluating our comparison criteria (section 2) we decided not to include mortality as one of the reported measures, as the mortality measure correlated very closely to our `failed treatment measure' (NTF).

7 Age Structure

The current implementation of our individual-based simulation is not intended to represent any particular country; however, we must choose a population age structure for the simulation. In the current implementation, we use the population age structure of Tanzania, obtained from the web site of the Tanzanian National Bureau of Statistics (see Table S5 below). The initial population size for each age 0 to 14 will be calculated based on these numbers; for other ages, the population size for each one-year age band is obtained by equally dividing the age classes below.

Age	Population	% of Total Population	Age Group	Population	% of Total Population
0	1,499,389	3.34%	15-24	8,562,875	19.06%
1	1,349,091	3.00%	25-34	6,302,172	14.03%
2	1,477,998	3.29%	35-44	4,340,066	9.66%
3	1,456,609	3.24%	45-54	2,716,946	6.05%
4	1,490,745	3.32%	55-64	1,544,557	3.44%
5	1,412,917	3.14%	>65	1,736,851	3.87%
6	1,420,161	3.16%			
7	1,394,553	3.10%			
8	1,279,389	2.85%			
9	1,152,017	2.56%			
10	1,340,272	2.98%			
11	951,527	2.12%			
12	1,443,723	3.21%			
13	1,022,836	2.28%			
14	1,034,229	2.30%			

Table S4. Population distribution of Tanzania

In 2013, the birth rate of Tanzania is estimated at 37.25 births per 1000 population per year. Note that with a birth rate of 37.25 births per 1000 population and the death rate as described in section 4, the population size of the simulation will double after 30 years.

8 Parasite Density Levels

(a) Asymptomatic Hosts

In a study of 314 asymptomatic children in Kampala, Uganda, Nsobya et al [40] showed that, at the enrollment point of the study when all children were asymptomatic, the asexual parasite densities had a range between 16 and 71840 parasites per microliter, with a geometric mean parasite density (GMPD) of 2630 parasites per microliter.

In a study from Gabon in 1995, 10 children aged from 5 to 11 with positive thick-film blood smears for *P. falciparum* and no symptoms for at least 5 days were followed daily for 7 days and then every 2 to 3 days until symptoms appeared [41]. The duration of the asymptomatic state for the 10 children ranged from 7 to 38 days and parasitaemia remained low (ranging from 10 to 10,000 parasites per microliter) during the asymptomatic state and usually increased with the appearance of symptoms (in 8 of 10 children).

However, some children had more than 2500 parasites per microliter for several days without developing symptoms. The appearance of symptoms, in all 10 children, was coupled with the occurrence of a new parasite genotype. An immunological hypothesis consistent with this observation is that the children had a moderate amount of specific immunity to their resident parasites, but this immunity was not strong enough to prevent a clinical episode caused by a newly acquired parasite. The short duration of asymptomatic parasitaemia in this study is likely due to the fact that the study was conducted in a high EIR area (EIR ≈ 50) where new infectious bites occur frequently.

Data from a study conducted in Madagascar from 1996 to 2005 show age-stratified histograms of parasite density among 541 asymptomatic *P. falciparum* malaria patients (Figure 5 in their paper) [42]. Across all age groups, the majority of asymptomatic cases had parasite densities lower than 500. A relatively high parasite density (>500parasites/μl) was observed in ~50% of children under ten and ~25% of individuals older than ten. A very high asymptomatic parasitaemia (>5000 parasites/μl) was observed in ~15% of under-tens and <2% of individuals over the age of ten.

In our simulation, we use 1000 parasites/μl as the initial parasite density for an asymptomatic individual. During the asymptomatic phase, parasite density decreases according to the host's level of immunity until it reaches 0.002 parasites per microliter which we define as the "cure level" where parasitaemia in the simulation is set to zero.

(b) Symptomatic Hosts

It is generally accepted that the total parasite burden for a symptomatic patient varies from 10^{10} to 10^{12} parasites [43]. Hence, for symptomatic adults and children we set the initial parasite density at 20,000 parasites/microliter (10^{11} total parasites), assuming that an adult individual has 5 liters of blood. For children the total parasite burden will be lower than 10^{11} parasites per symptomatic infection.

(c) Detection Level of Microscopy

A review of malaria diagnostic tools shows that the detection limit of microscopy has been estimated to be 4-20 parasites/microliter; however, in field conditions, a detection level of around 50-100 parasites/microliter is more realistic [44]. In our simulation, the detectable parasite density is chosen as 10 parasites/microliter and this threshold is used to determine the blood-slide prevalence, as shown in Figure 1 of the main paper.

9 Pharmacokinetics / Pharmacodynamics

Each individual is assigned a relative drug concentration value (relative to the initial concentration) for each drug that he or she has received during a treatment. We use a basic pharmacodynamic (PD) model to describe the exponential waning of drug concentration:

$$\mathrm{C}=\mathrm{C}_0 \cdot e^{-k_1 t}$$

where t is time in days, k_1 is the elimination rate constant, and C_0 is the maximum initial concentration after dosing. When an individual is treated, the initial drug concentration C_0 will be drawn from a normal distribution with mean of one and standard deviation ranging from 0.1 to 0.4; C_0 will be constrained to be within the range [1-3*sd, 1+3*sd]. By adjusting the value of the standard deviation, we can vary the efficacy of a particular drug.

The relationship between k_1 and drug half-life is given by the following equation:

$$k_1 = \frac{\ln 2}{t^*}$$

where t^* is the half-life of the drug.

To formulate the pharmacokinetic (PK) model, a modified version of the standard Michaelis-Menton equation (which is known as Hill's equation) was used [45]:

$$percent\_p\_remove\_per\_day = p(C) = p_{max} \cdot \left(\frac{C^n}{C^n + EC_{50}^n} \right)$$

is the fraction of parasites that are killed in a given day. The value C is the current drug concentration, p_{max} is the maximum fraction of parasites that can be removed by a drug in one day, EC_{50} is the concentration level corresponding to 50% effect, and n is the slope of the concentration-effect curve. In the simulation, the parasite density is updated according to the following equation:

$$P(t) = \big(1 - p(C)\big) \cdot P(t-1).$$

We can consider the term $(1 - p(C))^{-1}$ as the parasite reduction ratio and $(1 - p_{max})^{-1}$ as the initial parasite reduction ratio. In the simulation, estimated *in vivo* PRR values of different drugs, as shown in Table 1 of [21], correspond to the following p_{max} values for the following antimalarial drugs:

**Antimalarial drugs**	**PRR/day**	p_{max}
Artemnisinin, artesunate, artemether	10^2 – 10^2.5	0.99 – 0.9968; see also [46] for a higher estimate
4-Aminoquinoline (Cholorquine, Amodaquine), halofantrine	10 – 10^2	0.9 – 0.99
Quinine, mefloquine, sulfadoxine-pyrimethamine	10^0.5 – 10^1.5	0.68 – 0.9684
Antimalarial antibiotics (doxycycline, clindamycin), desferrioxamine	10^0.35 – 10^0.5	0.55 – 0.6838

Table S5: Maximum parasite killing rates for different antimalarial drugs.

Drug half-life values were obtained from a 2001 WHO Report on antimalarial drug use [47] and several other sources (shown in the table below).

**Drug**	**Half-life (t^*)**
Chloroquine	10 days
Amodiaquine	9 (7-12) days [48]
Sulfadoxine	8days
Pyrimethamine	4 days
Proguanil	16 hrs
Quinine, quinidine	10-12 hrs
Mefloquine	10-40 days
Halofantrine	1-6 days
Artemisinin derivatives	4-11 hrs (DHA: 11-12 hrs)
Lumefantrine	3-6 days [49]
Piperaquine	33 days [50]

Table S6: Half-lives of antimalarial drugs.

To find specific values of n and EC50 for a particular drug or drug combinations, we fixed the drug half-life and p_{max} value which are known, and we adjusted n (ranging from 10 to 25), EC50 (ranging from 0.4 – 0.6), and the standard deviation of the initial drug concentration until the PK/PD profile of that drug or combination corresponded to the treatment efficacy measured from clinical trials. For example, for artemether-lumefantrine, we set a half-life of zero days (i.e. drug is completely out of the system on the following day), n=25, EC_{50}=0.6, $p_{\max}$=0.999 [46] for artemether, and a half-life = 4.5 days, n=21, EC_{50}=0.6, $p_{\max}$=0.99 for lumefantrine. This corresponded to a treatment efficacy of 94.9%.

10 Model of Immunity and Symptoms

Using M as the variable describing the general immune level ($0 < M < 1$), the equation for immune acquisition while a host maintains a blood-stage infection is

$$1 - M(t_2) = \big(1 - M(t_1)\big)\, e^{-a_2(a)\cdot(t_2 - t_1)},$$

where the rate of immune acquisition (a_2) increases with age. We model this age dependency by allowing the rate a_2 to increase by 1% every year for hosts over the age of ten. For hosts under the age of ten, we use a convex function to describe the slower immune acquisition observed in children. For children under ten ($1 < a < 10$), the age-specific immunity acquisition rate is

$$a_2(a) = (a/10)^{\kappa}\; \times (1.01)^{a} \times\; 0.00125\,,$$

where a is treated as an integer and κ determines the convexity of the function (this is fit from figures S3 and S4 below). Individuals younger than six months have maternal immunity (see below) with no immune acquisition; individuals between 6 and 12 months no longer have maternal immunity and acquire immunity when infected at a rate a_2 that is half as fast as the rate for one-year olds. The parameter κ sets the convexity of age-specific immune acquisition – for low κ the immune-acquisition rate of children quickly reaches the normal level observed in adults, and for high values of κ children will have slow immune acquisition rates until they reach an age of nine or ten. We varied κ between 0.1 and 5.0 to determine how it affected rates of clinical presentation, and in our simulations we use $\kappa = 1.0$.

For individuals older than ten, the immune acquisition rate is

$$a_2(a) = (1.01)^{a-10}\; \times\; 0.0013809\,.$$

Using these parameters, a naïve ten-year old child would reach a state of 80% immunity (M=0.8) in about three years. A naïve 30-year old adult would reach this state after about 2.5 years. Assuming κ=1.0, a persistently infected two-year old would reach 80% immunity by age nine, and a persistently infected five-year old would reach 80% immunity by age ten.

Immunity wanes according to the equation

$$M(t_2) = M(t_1)\, e^{-a_1(t_2 - t_1)}$$

where a_1 is set to 0.0025. This corresponds to 90% immune loss after 2.5 years [15]. Newborns are given maternal immunity (M=1) which wanes linearly over six months and is then set to zero.

The level of immunity M determines the duration of parasitaemia (see section 4) and the probability that a new infectious bite leads to symptomatic/clinical malaria. Our model most closely resembles the immunity model in Filipe et al. [15], which describes two types of immunity in individuals that are acquired and lost at different rates. In our model, there is a single immunity variable but this variable M determines two clinical phenotypes (probability of developing symptoms and rate of parasite clearance) that are acquired and lost at different rates as they are non-linear functions of M.

Note that the behavior and structure of our immunity model will not exactly mirror that of the 2007 Filipe model, as their model is compartmental and immunity is modeled as a population-level variable. For example, in our model a naïve individual can become infected and acquire a higher degree of immunity, associated with faster clearance, over the 6-month duration that he/she is clearing parasites; but in a compartmental model with a population-level immunity variable this host would remain naïve and a slow-clearer as long as the EIR and prevalence in the population remained low.

The probability of progression to symptoms, as a function of M, is modeled as

$$P_{clin} = \frac{0.99}{1 + (M/M_{\text{mid}})^z}$$

The parameter z describes the relationship between the level of immunity and the likelihood of developing symptoms, and z is varied between 2.0 and 8.0 to determine its effects on patterns of clinical malaria; we set M_{mid} to 0.4 as in the Filipe paper [15].

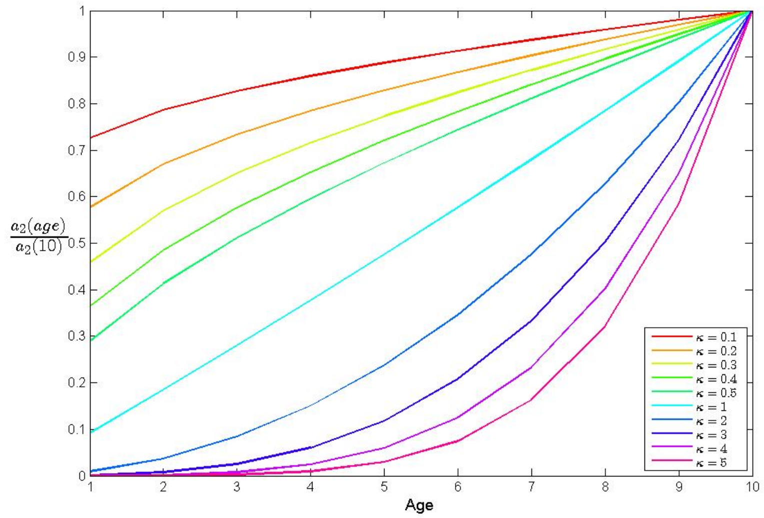

Figure S1: The y-axis shows a child's relative immune acquisition rate when compared to a ten-year old. Ten different lines are drawn for different values of the κ parameter ranging from 0.1 to 5.0.

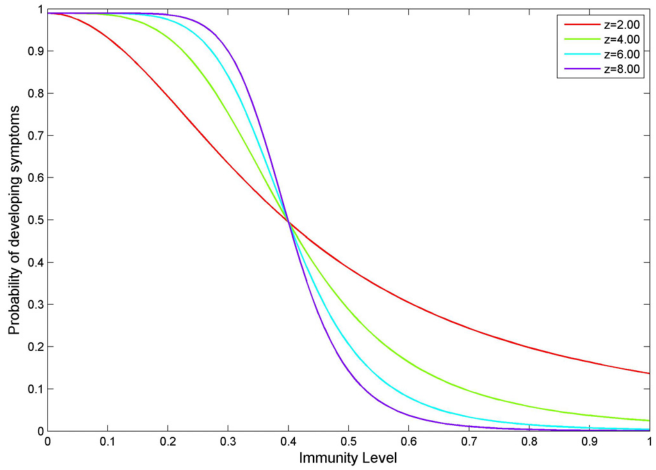

Figure S2: The probability of progressing to clinical disease after in infectious bite, based on host's immune level and the parameter z.

11 Incidence of Clinical Episodes by Age

Because there is no single measure of immunity, and because some of the unknown parameters from section 8 (a_1, z, and κ) are impossible to measure directly, we perform a model validation exercise to determine if the age-stratified annual incidence of clinical episodes in the simulation matches what is observed in the field.

If immune acquisition is rapid and if immunity has a strong effect on protecting individuals from severity/symptoms, then the age distribution of clinical cases should have a spike in the younger age groups with a rapid drop off as age increases. If immune acquisition is slow with weak effects of symptoms prevention, the age-distribution of clinical cases would look flat in low-transmission areas and nearly-flat (slowly decreasing with age) in higher transmission areas. Essentially, as EIR increases children acquire immunity more quickly, and a larger fraction of clinical episodes should be observed in the younger age classes.

We use eight sites (from five publications) that report incidence patterns of clinical episodes by age for varying levels of EIR. The table below shows the annual incidence of clinical malaria episodes for 2-year olds, 10-year olds, and 17-year olds for these eight sites. These age groups were chosen for convenience based on the available data. The numbers in the table are approximations as it is only necessary to see roughly how these numbers change relative to one another.

		Clinical Episodes Per Year		
		Age		
Study Reference	EIR	2	10	17
Mwangi et al[16]	10	1.60	0.50	0.35
Saute et al [17]	18	0.42	0.13	no data
Smith et al [20]	20	2.20	2.80	1.00
Ghani et al [18]	30	2.10	2.70	1.20
Mwangi et al [16]	37.5	1.55	0.30	0.25
Guinovart et al[19]	38	0.87	0.28	no data
Ghani et al [18]	200	5.00	0.60	0.30
Smith et al [20]	200	5.00	0.50	0.20

Table S7. EIR and clinical episodes/year by for ages 2, 10, and 17, extracted from 8 referenced studies.

Figures S3 and S4 show the ratio, from the table above, of clinical episodes in 2-year olds to clinical episodes in 10-year olds. These data are compared to our simulation output.

Simulation results show that the ratio of clinical episodes in age-group 2 to age-group 10 has a positive log-linear relationship with EIR. The combination $\kappa = 1$ and $z = 4$ was the closest match to the data points shown in the last panel of Figures S3 and S4, and we fix $\kappa = 1$ and $z = 4$ in our simulations. We also verified that the EIR-prevalence relationship (section 13) and EIR-multiplicity of infection relationship (section 15) were not sensitive to the values of z and κ.

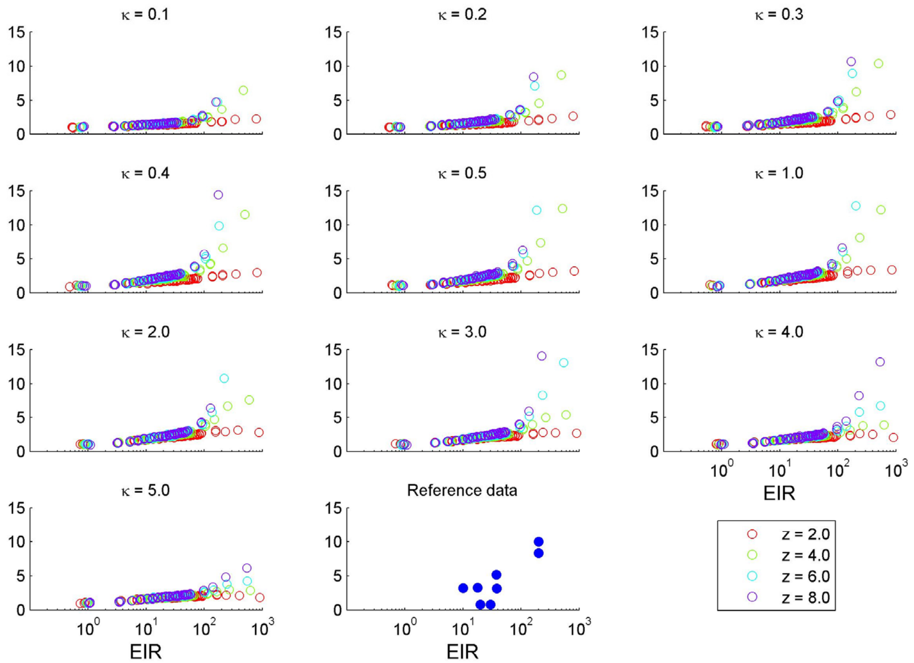

Figure S3: The *y*-axis shows the ratio of the number of annual clinical episodes in 2-year olds to the number in 10-year olds. Ten simulations were done for each combination of *κ* (different panels) and *z* (different colors). The last panel shows the data from Table S7. In these simulations the treatment coverage was *f* = 0.0.

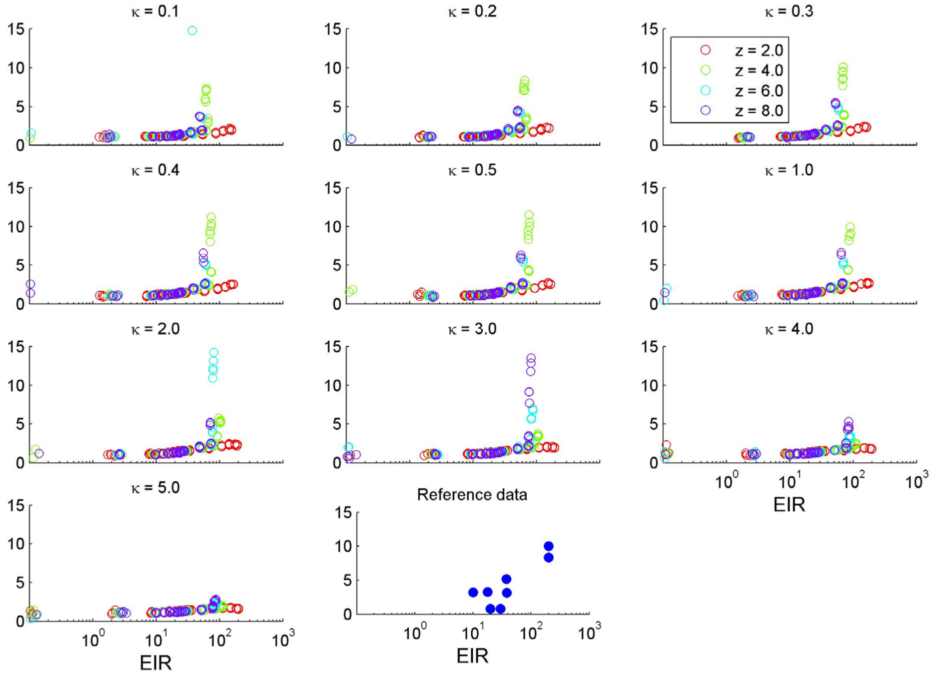

Figure S4: The *y*-axis shows the ratio of the number of annual clinical episodes in 2-year olds to the number in 10-year olds. Ten simulations were done for each combination of *κ* (different panels) and *z* (different colors). The last panel shows the data from Table S7. In these simulations the treatment coverage was *f* = 0.5 with a drug with a 7-day half-life.

After choosing a particular immune acquisition behavior for our model (κ = 1 and z = 4), we test whether the model reproduces the expected age-specific clinical pattern under different transmission scenarios. The burden of clinical disease should shift from the older age-group to the younger age-groups as transmission rate increases, and this is indeed what we observe in Figures S5 (no treatment) and S6 (50% treatment coverage).

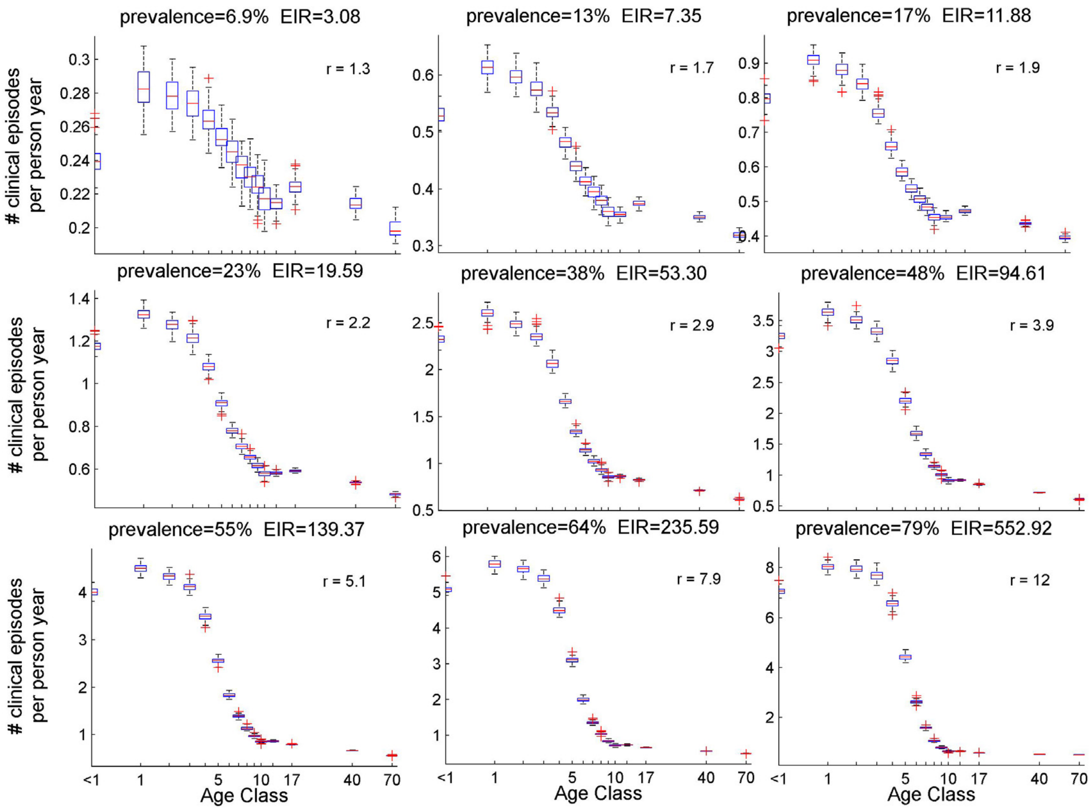

Figure S5: The age-specific clinical pattern under different transmission intensity (EIR increases from left to right and from top to bottom). The *r*-values in the top right corner show the ratio of clinical episodes ratio between two-year olds and ten-year olds in the simulation. Boxplots show medians and interquartile ranges from 100 simulations. All simulations run with κ = 1 and z = 4. In these simulations the treatment coverage was f = 0.0.

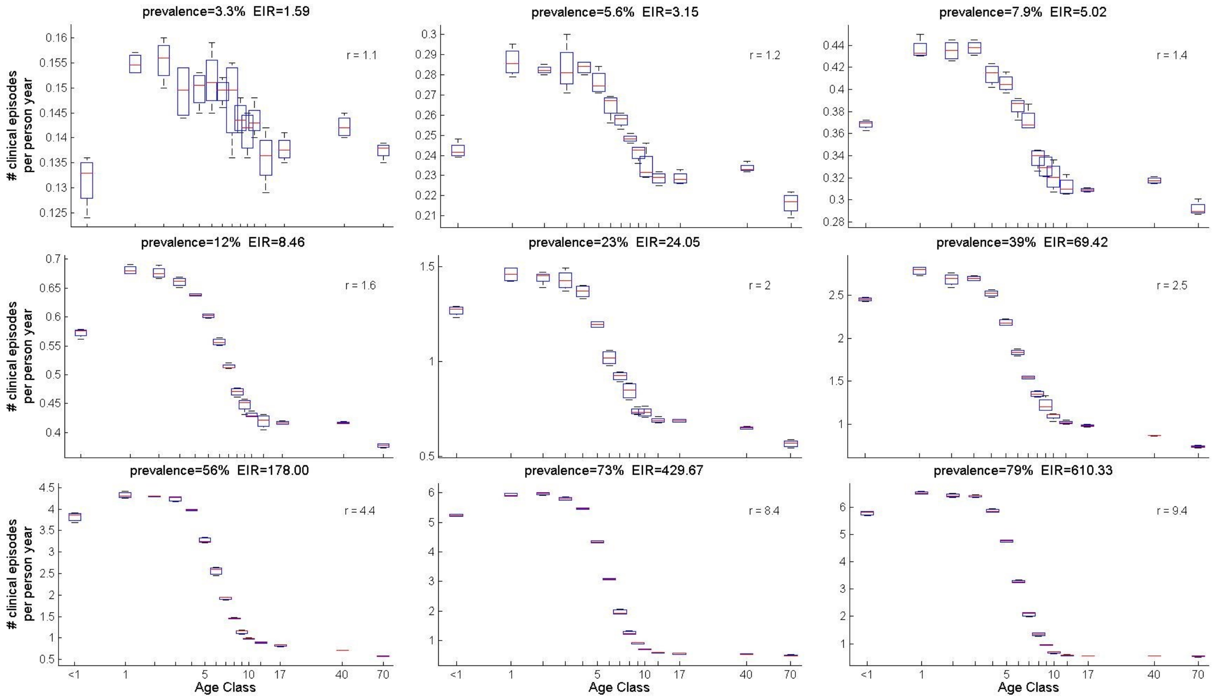

Figure S6: The age-specific clinical pattern under different transmission intensity (EIR increases from left to right and from top to bottom). The *r*-values in the top right corner show the ratio of clinical episodes ratio between two-year olds and ten-year olds in the simulation. Boxplots show medians and interquartile ranges from 100 simulations. All simulations run with $\kappa = 1$ and $z = 4$. In these simulations the treatment coverage was $f = 0.5$ with a drug with a 7-day halflife.

12 Prevalence of Symptoms and Blood-Slide Prevalence (φ value)

For all age classes, we are interested in the ratio of symptomatic cases over all infected cases (what we call the "φ value") as this will give us a measure of the selection pressure on the parasite population as only symptomatic cases are treated. We would expect that for φ decreases as EIR increases, as in the model described by Boni et al [8]. Very few data exist on this measurement. A recent review on asymptomatic parasitaemia and transmission showed that φ ranges from 0.40 to 0.01, as prevalence increases from 1% to 85% [51]. Yekutiel reports φ as being between 0.06 and 0.17 [52]. In two other studies, it appears to be below 0.20 [53,54]. Note that this ratio is very sensitive to the denominator, which is very sensitive to the detection method used in each study.

In our simulations, the range of φ does stay below 0.20, and φ decreases with EIR. However, we also observe a complicated interaction between φ and age. As shown in Figures S7 and S8 below, the φ-value for younger age groups (<10) increases as EIR increases; the explanation for this is that in high transmission areas, children are bitten more often and experience symptoms more often.

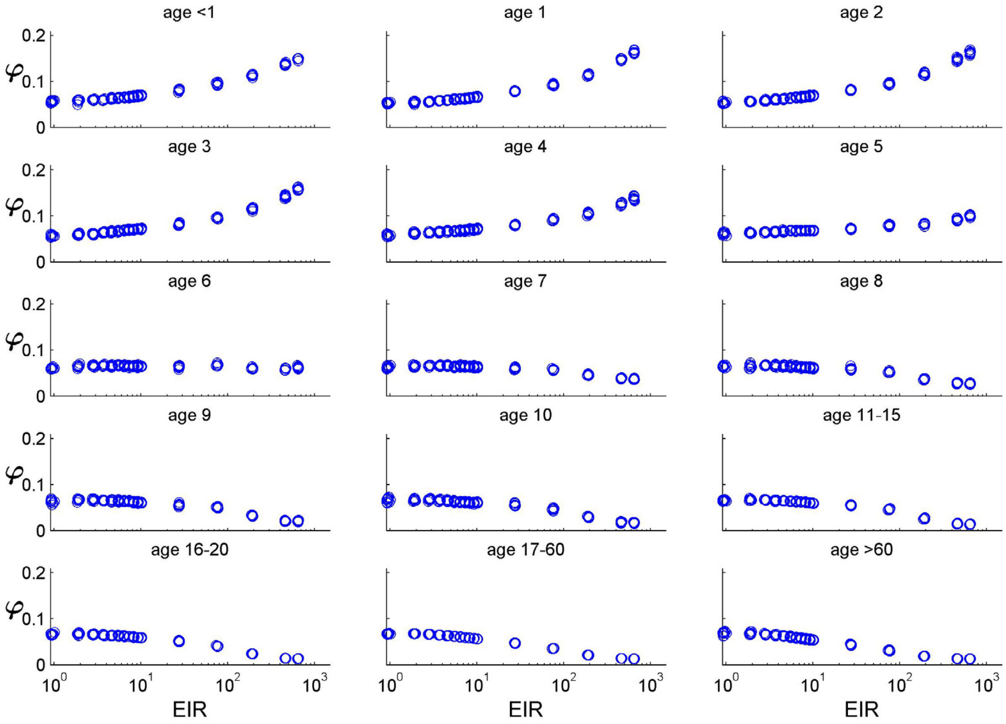

Figure S7: 400 simulations were run to equilibrium at EIR levels ranging from 0.6 to 550. In these simulations treatment coverage was set to f = 0.0. Immune acquisition parameters: κ = 1 and z = 4.

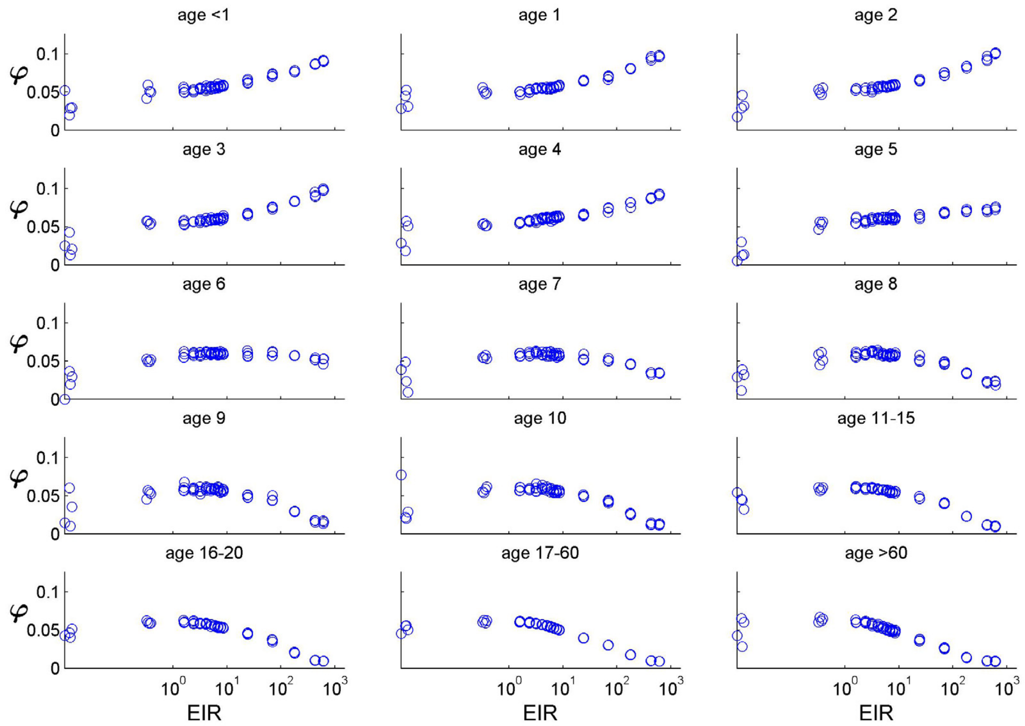

Figure S8: 400 simulations were run to equilibrium at EIR levels ranging from 0.14 to 550. Treatment coverage was set to f = 0.5, with a single 7-day half-life drug of approximately 80% efficacy. Immune acquisition parameters: κ = 1 and z = 4.

13 Relationship between EIR and Malaria Prevalence

It is well known that the relationship between the entomological inoculation rate (EIR) and malaria prevalence (PR) should be non-linear, as prevalence will saturate close to 100% as EIR increases. Beier et al (1999) reviewed data from 31 sites in Africa and suggested a linear relationship between log(EIR) and prevalence (their Figure 1) [55]. Similarly, Hay et al [56] analyzed data from 22 countries across Africa between 1980 and 2004 and also found the log-linear relationship between EIR and PR. Figures S9 and S10 show this relationship for our model. Saturation at high EIR is seen as expected, and a general log-linear relationship (bottom panel) is seen between EIR and prevalence.

As a second validation, we consider an EIR-PR relationship, proposed by Smith et al (2005), which includes biting rate heterogeneity [3]. The model:

$$PR = 1 - \left(1 + \frac{b\varepsilon}{rk}\right)^{-k}$$

where ε is EIR, b is the probability that an infectious bite results in an infection, r is the host's clearance rate, and $1/k$ is the variance of a unit-mean gamma distribution describing heterogeneity in biting rates. This model fit the data [56] better than five other models they proposed.

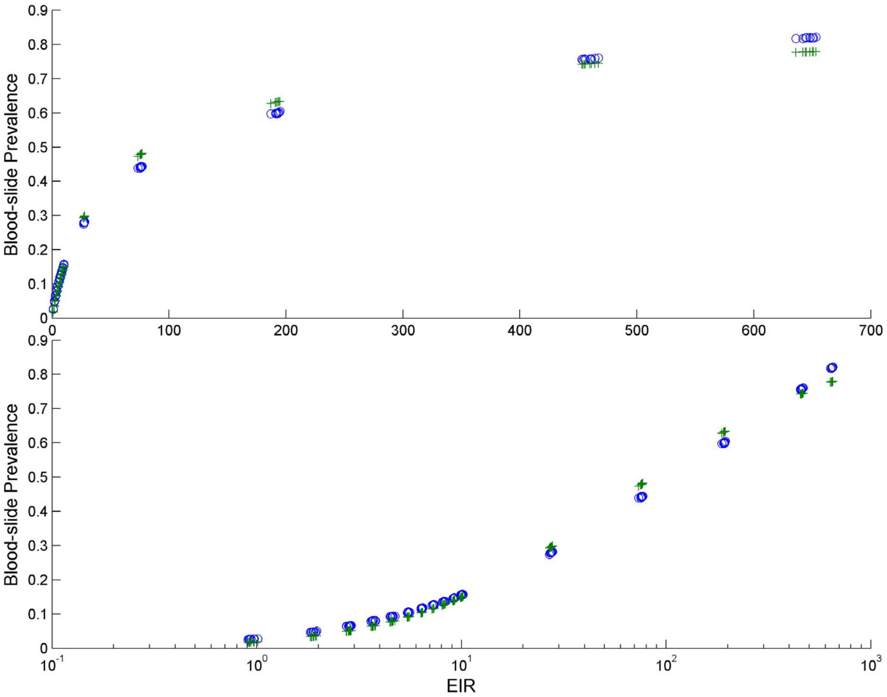

Figure S9: Model output shown in blue circles. Relationship between EIR and blood slide prevalence shown on linear scale (top) and log scale (bottom). The model was run with Γ-distributed relative biting rate with a coefficient of variation equal to 2.0. Treatment coverage is f = 0.0. The green crosses represent the fitted data from the Smith et al model with b/r = 0.0194, $1/k$ = 0.447 [3].

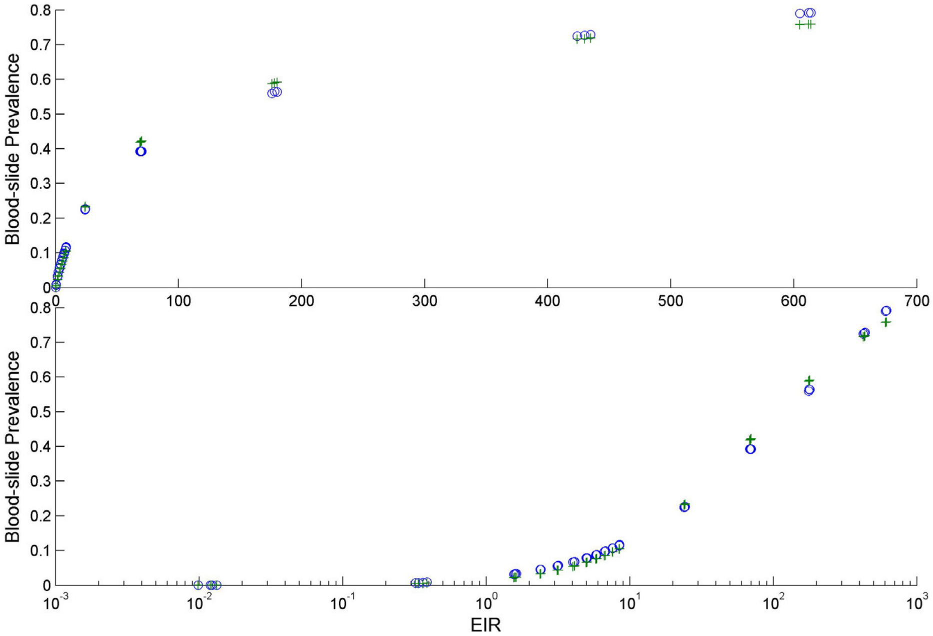

Figure S10: Model output shown in blue circles. Relationship between EIR and blood slide prevalence shown on linear scale (top) and log scale (bottom). The model was run with Γ-distributed relative biting rate with a coefficient of variation equal to 2.0. Treatment coverage is f = 0.5 with a single 7-day half-life drug of approximately 80% efficacy. The green crosses represent the fitted data from the Smith et al model with b/r = 0.0194, $1/k$ = 0.447 [3].

14 Prevalence by Age

As discussed in previous sections, it is believed that immunity is acquired slowly in the 2-10 age group, with individuals over the age of ten acquiring immunity (as a result of parasitaemia) at approximately the same rate. A result of this assumption is that older age groups should have faster clearance rate and lower prevalence compared to younger age groups, as is observed in the field [11]. A decrease in transmission or prevalence may lead to a shift in peak risk of malaria infection to older age groups [57–59]. Age-specific prevalence data, summarized by Smith et al [60], show the shift in peak prevalence from older age groups to younger age groups as transmission increases. In a hypoendemic area, the prevalence appeared to be distributed equally across all the age groups. Our model exhibits the expected age-specific prevalence patterns as shown in Figures S11 and S12 below.

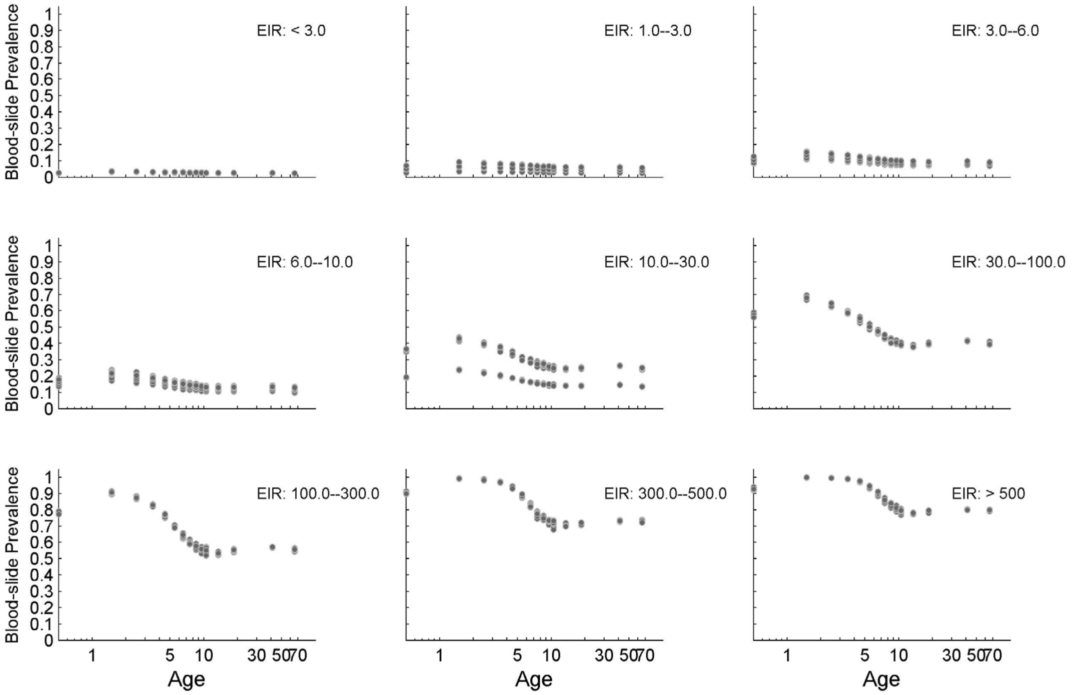

Figure S11: The graphs shows age-specific blood-slide prevalence for different transmission intensities; $\kappa = 1$, z = 4, treatment coverage $f = 0$.

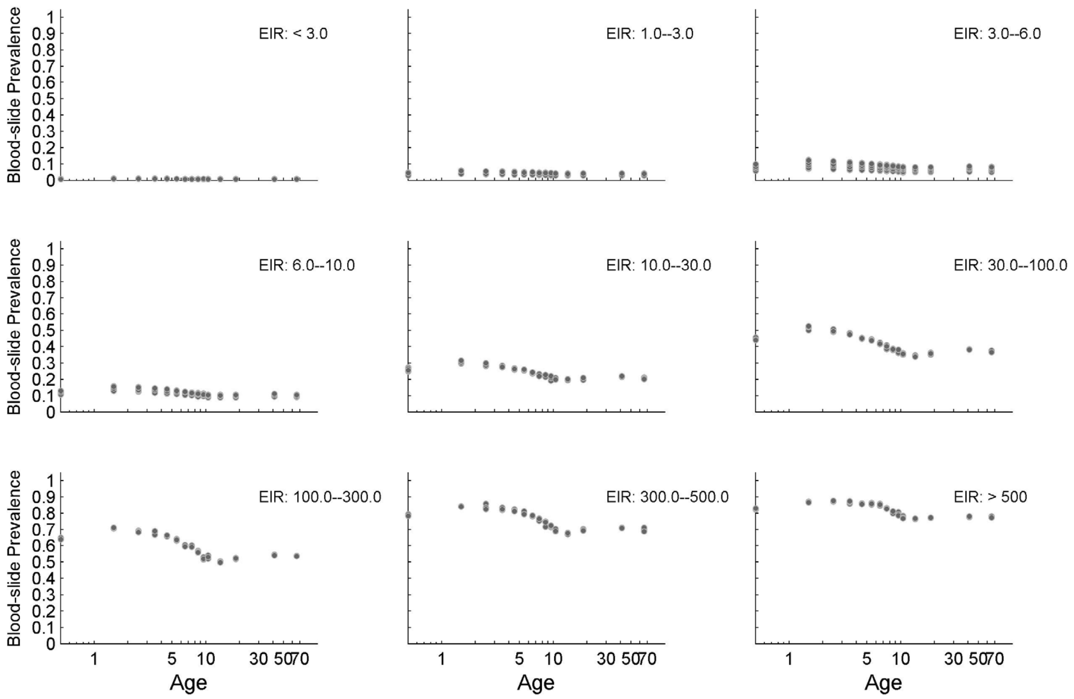

Figure S12: The graphs shows age-specific blood-slide prevalence for different transmission intensities; κ = 1, z = 4, treated coverage f = 0.5, with a single 7-day half-life drug of approximately 80% efficacy.

15 Multiplicity of Infection

The multiplicity of infection (MOI) data from Owusu-Agyei et al [61] is used to validate the distribution of the number of parasite clones within each host. The EIR in the Owusu-Agyei study is estimated at approximately 300 bites per person year. We generate two model scenarios with this high level of transmission (Figures S13 and S14) and plot the multiplicity of infection (MOI) distribution from the equilibria reached in these simulations, alongside the data from the Owuse-Agyei paper (green squares below).

The shape of MOI distribution depends on the transmission intensity. In a low-transmission setting, most infected individuals have a single parasite clone present in their blood, while in high-transmission areas the majority of individuals have multiple clones present.

Arnot [62] showed a relationship between EIR and the average number of malaria parasite clones per infection with a positive linear association on a log-log scale. Our model output shows a similar pattern (not shown here), but there are simply too few data points from the field on the EIR-MOI relationship to determine what the null shape of this distribution should be.

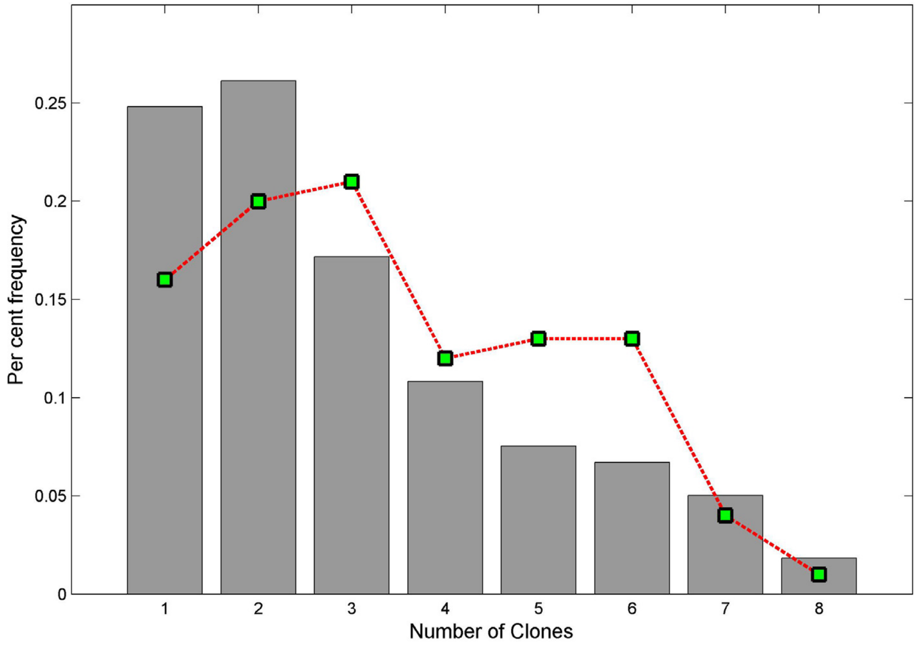

Figure S13: Distribution of number of clones per infection. The bar graph shows the output from our simulation with EIR = 290 while the dashed line (green squares) represents the data from Owusy-Agyei's study with EIR approximately 300 (κ = 1, z = 4, f = 0.0).

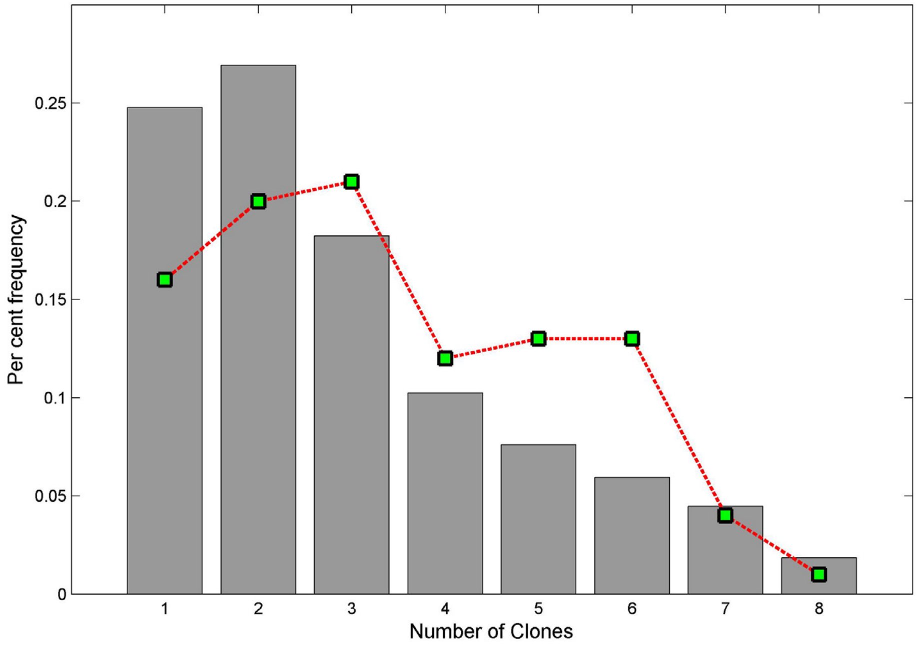

Figure S14: Distribution of number of clones per infection. The bar graph shows the output from our simulation with EIR = 335 while the dashed line (green squares) represents the data from Owusy-Agyei's study with EIR approximately 300 (κ = 1, z = 4, f = 0.5, with a single 7-day half-life drug of approximately 80% efficacy).

16 Biting-Rate Heterogeneity and Prevalence

Heterogeneity in transmission was modeled by assigning a relative biting level (b_i) to each host, which describes that host's relative attractiveness to mosquitoes. The higher an individual's relative biting level, the more frequently mosquitoes will bite that individual. A convenient and classic formulation of transmission heterogeneity is the "20/80 rule" [63] which states that in many epidemiological scenarios with transmission heterogeneity 20% of the population is responsible for approximately 80% of transmission.

The relative biting levels (b_i) in our simulation follow a gamma distribution. In the Smith et al paper [3], the inferred coefficient of variation (mean 1, variance 4.2) is approximately 2.05, and we set this coefficient of variation as our default. Sensitivity analyses (section 17) are conducted with coefficients of variation set to 1.0 and 2.0.

The effect of the standard deviation on the 20/80 rule is shown in the graphs below.

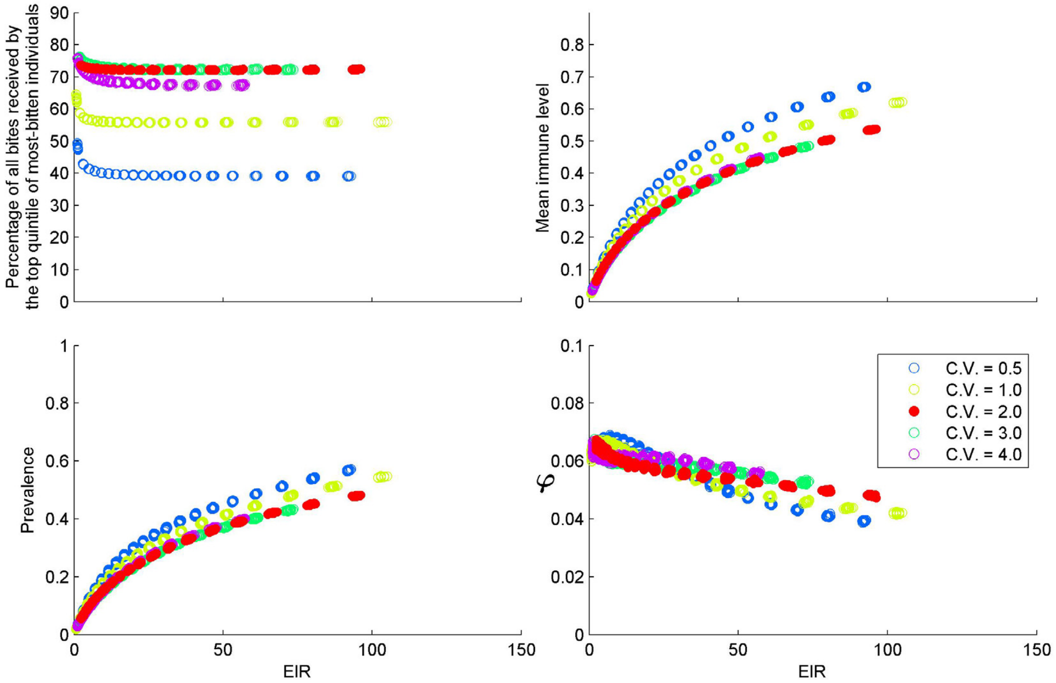

Figure S15: The sensitivities of four key model relationships to the standard deviation in relative biting rate. Treatment coverage *f* is set to 0.0.

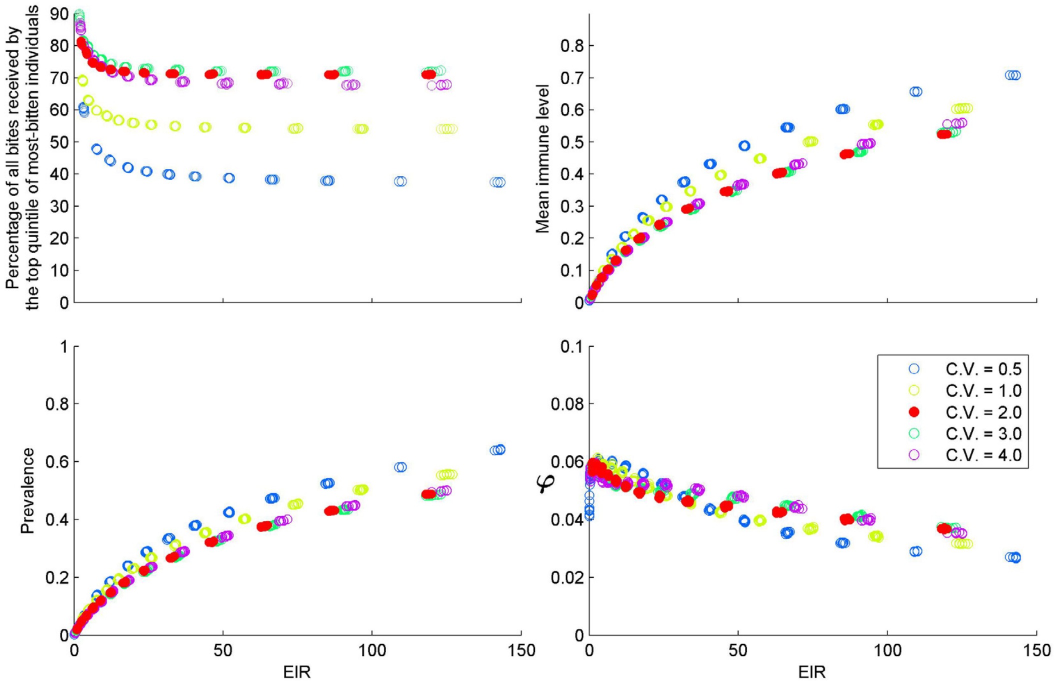

Figure S16: The sensitivities of four key model relationships to the standard deviation in relative biting rate. Treatment coverage *f* is set to 0.5.

17 General Sensitivity Analysis

To perform a sensitivity analysis to certain epidemiological, pharmacological, evolutionary parts of our model, we varied the parameters/scenarios below.

First we varied the transmission setting by varying the transmission coefficient β (four different values) as well as the coefficient of variation in the biting rate (two different values). The equilibrium behaviors of these eight scenarios are described below. In these test scenarios, we assume that 50% of clinical cases are treated with a drug of 80% efficacy and a 7-day half-life.

Transmission Coefficient (β)	Coefficient of Variation in Biting Rate	Prevalence at Equilibrium	Annual EIR at Equilibrium
0.50	2.0	18.7%	18.0
0.50	1.0	23.1%	20.3
0.25	2.0	9.17%	6.20
0.25	1.0	9.20%	5.43
0.20	2.0	6.56%	4.07
0.20	1.0	4.88%	2.36
0.14	2.0	2.70%	1.33
0.14	1.0	0.01%	<0.01

Because the last scenario in the table above resulted in frequent extinction, this transmission setting was not used in the sensitivity analysis. Only the first seven transmission settings were used. Note that in the simulations, a different EIR will be observed as ACTs are used in our simulated scenarios and the equilibrium prevalence and EIR are lower than in the table reported above. The table above can be thought of as the "pre-ACT era" transmission setting.

The treatment coverage was varied across the five values f = 0.5, 0.6, 0.7, 0.8, and 0.9.

The cost of resistance was varied across the three values c_R = 0.001, 0.005, and 0.010.

To vary the effect of the drug concentration in the probability of drug-resistance evolution we varied the parameter k (see "Drug Resistance Evolution" heading in section 1). Three values are used, k = 0.5, 2.0, and 4.0.

The drug half-lives were varied to test four different scenarios: (a) all three partner drugs have a half-life of 4.5 days, (b) all three partner drugs have a half-life of 9 days, (c) all three partner drugs have a half-life of 28 days, (d) the three partner drugs have three different half-lives of 4.5 days (corresponding to lumefantrine), 9 days (corresponding to amodiaquine), and 28 days (corresponding to piperaquine).

In each of the above scenarios, it was assumed that three ACTs were available, and three strategies were evaluated: MFT, 5-Year Cycling, and Sequential Deployment.

Thus, a total of 3780 scenarios are simulated. This requires approximately 4-5 days of computation time on a modern 70-core computer cluster. A total of five complete sensitivity analyses were run (18,900 simulations), and the results are summarized below.

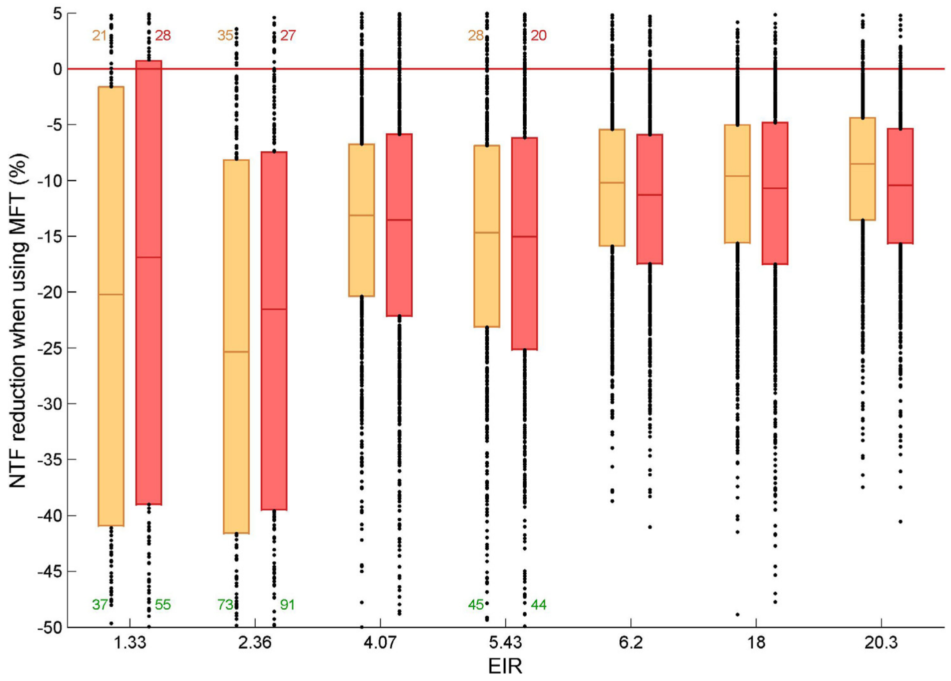

Figure S17. The boxplots show the percent reduction in NTF achieved by using an MFT policy, when compared to sequential deployment (orange) and five-year cycling (red), across all parameter combinations in the sensitivity analysis. The boxes show interquartile ranges, stratified by different transmission intensities. For each parameter combination in the comparison, only scenarios with no extinction (NTF>0.25) are included in the calculation of interquartile ranges. The numbers at the top and bottom of each boxplot show the number of comparisons for which one strategy caused extinction but the comparator strategy did not. The green numbers indicate the number of simulations for which MFT resulted in extinction but the cycling/sequential strategy did not, while the red and orange numbers show the number of simulations when the cycling (red) or sequential (orange) strategies reached extinction but MFT did not. There are fewer extinction under EIR=4.07 than EIR=5.43, because the former is associated with a higher coefficient of variation in the mosquito biting rate. Across the different transmission settings and counting all simulations (including those that did and did not lead to extinction) NTF numbers are lower for MFT than other strategies (all $p < .002$; Mann-Whitney).

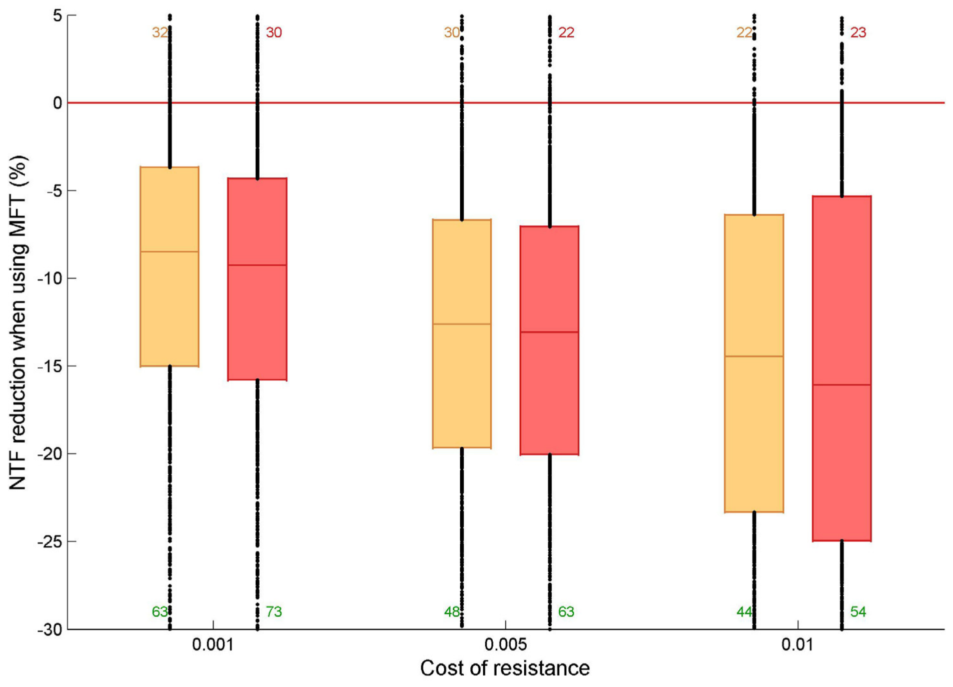

Figure S18. The boxplots show the percent reduction in NTF achieved by using an MFT policy, when compared to sequential deployment (orange) and five-year cycling (red), across all parameter combinations in the sensitivity analysis. The boxes show interquartile ranges, stratified by different costs of resistance. For each parameter combination in the comparison, only scenarios with no extinction (NTF>0.25) are included in the calculation of interquartile ranges. The numbers at the top and bottom of each boxplot show the number of comparisons for which one strategy caused extinction but the comparator strategy did not. The green numbers indicate the number of simulations for which MFT resulted in extinction but the cycling/sequential strategy did not, while the red and orange numbers show the number of simulations when the cycling (red) or sequential (orange) strategies reached extinction but MFT did not. For the three different costs of resistance, and using all simulations (including those that did and did not lead to extinction), NTF numbers are lower for MFT than other strategies (all $p < 10^{-6}$; Mann-Whitney).

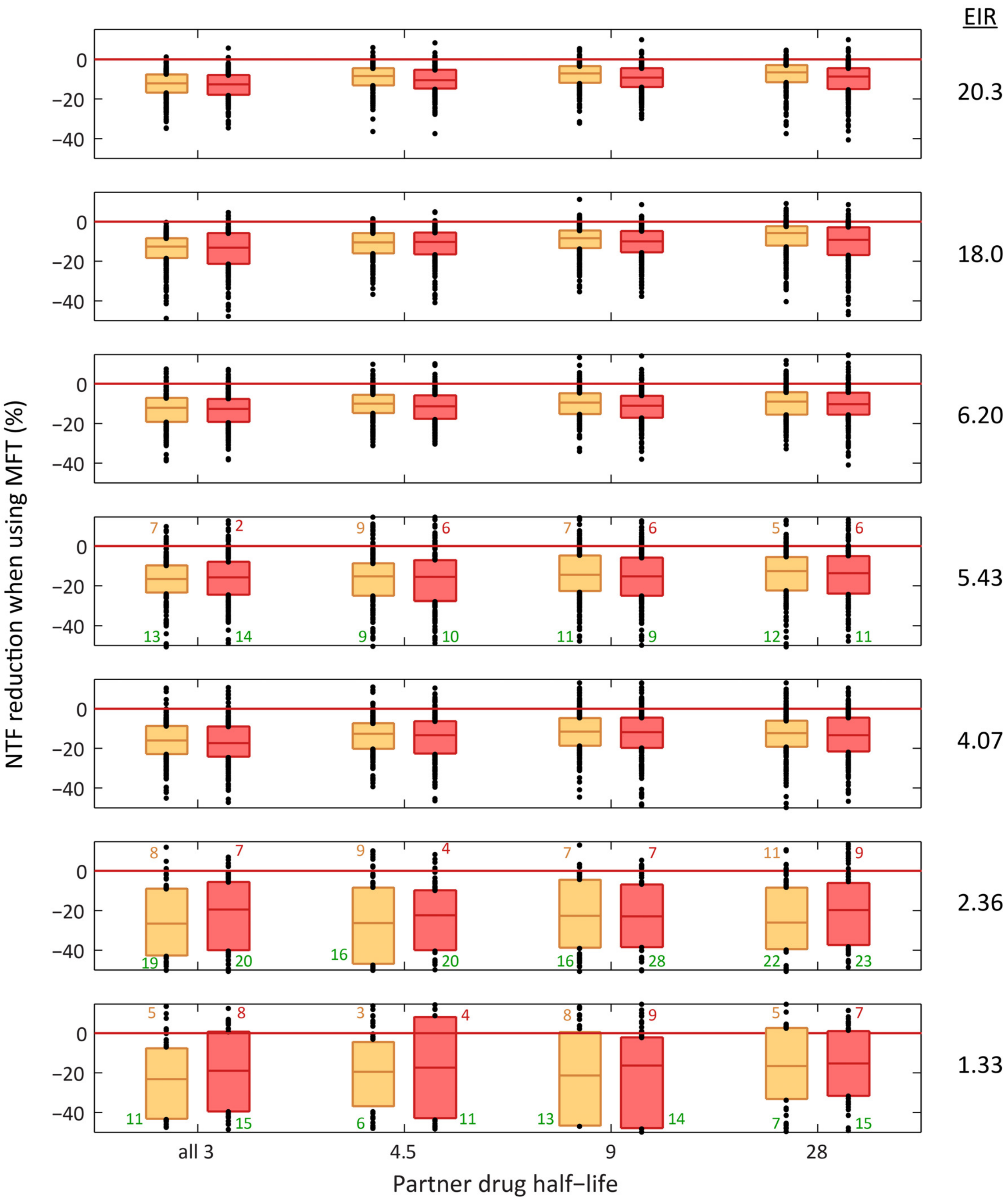

Figure S19. As figures S17 and S18, stratified by EIR and partner drug half-life. For the higher transmission scenarios (top five rows), NTF numbers are lower for MFT than other strategies ($p < 10^{-4}$; Mann-Whitney, for each boxplot). For the bottom two rows, the main qualitative feature of these comparisons is the number of total extinctions in the simulations (approximately 50%) and not a comparison of medians or ranks. MFT caused more extinctions than Cycling or Sequential Deployment in all of these comparisons; for simulations that did not result in extinction the NTF values for MFT were lower ($p < .002$; Mann-Whitney, for each boxplot).

18 Additional Figures for Specific Sensitivity Analyses

Varying the parameter *k* which describes the relationship between drug concentration and mutation probability.

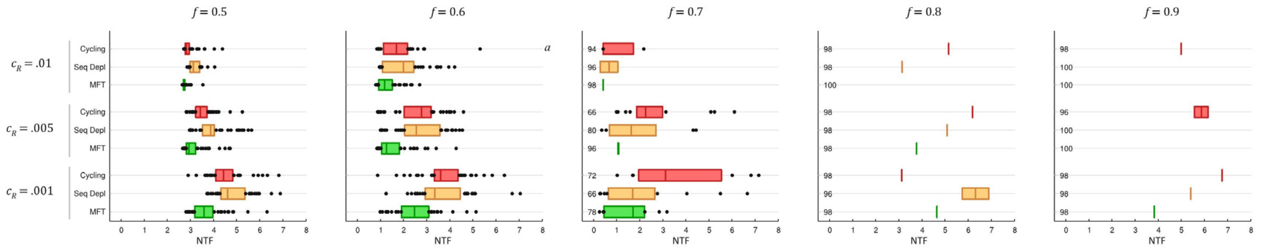

Figure S20. As Figure 2 of the main text, but with k = 0.5; i.e. the probability of a drug-resistance mutation is proportional to drug concentration. Fifty simulations are shown for each strategy, drug coverage (f), and cost of resistance (c_R) combination. NTF values exhibited a bimodal distribution corresponding to simulation runs that did or did not result in extinction. Box plots show NTF interquartile ranges for simulations with no extinctions. The numbers on the left-hand side of each plot show the percentage of simulations that resulted in extinction. Mutations accumulate more slowly for k=0.5 (this figure) than for k=4.0 (Figure 2, main text), and for this reason extinction is more likely for k=0.5 when treatment coverage is very high. For f = 0.5 and f = 0.6, NTF values were lower under MFT (p=0.02; except for comparison a).

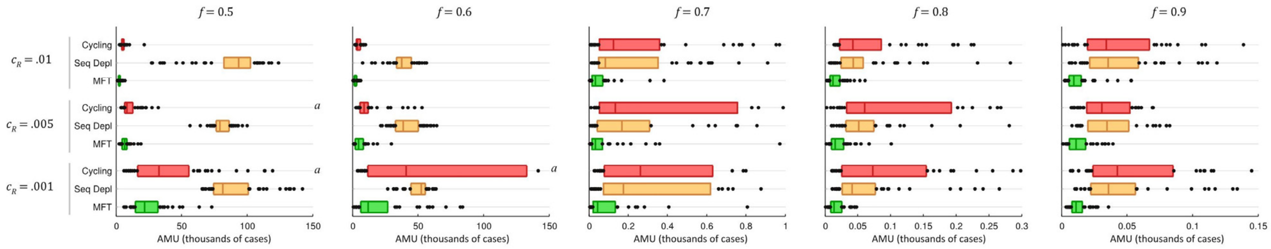

Figure S21. As Figure 3 of the main text, but with k = 0.5; i.e. the probability of a drug-resistance mutation is proportional to drug concentration. Fifty simulations are shown for each strategy, drug coverage (f), and cost of resistance (c_R) combination. AMU values did not exhibit a bimodal distribution, and interquartile AMU ranges are shown as boxplots for all fifty simulations. For $f \geq 0.7$, AMU values were lower under MFT ($p < 0.003$). For $f \leq 0.6$, AMU values were lower under MFT ($p < 0.01$, except for those labeled a); note that in these three cases it was only the 25th percentiles were not statistically different.

Varying the transmission setting.

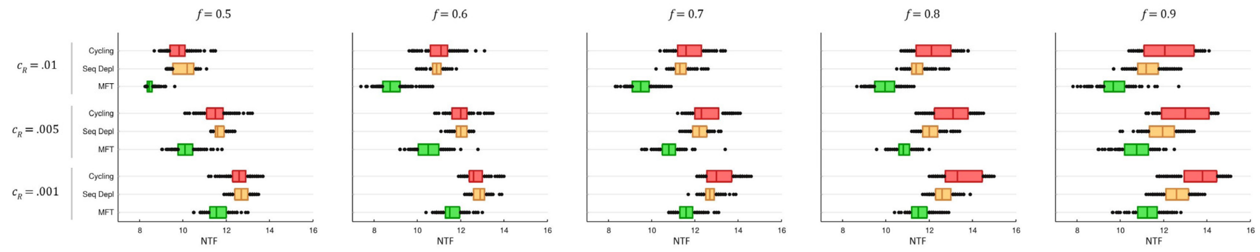

Figure S22. As Figure 2 of the main text but in a moderate transmission setting corresponding to the fifth scenario listed in the table on page 27. Annual EIR is 4.07. All $p < 10^{-10}$ when comparing MFT to other strategies. Each boxplot corresponds to 100 simulations.

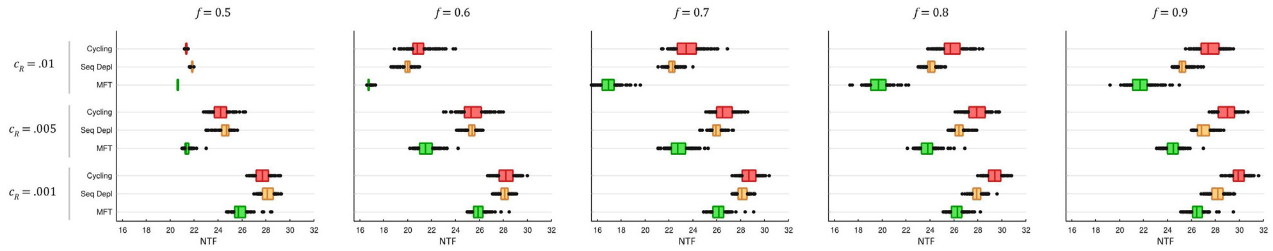

Figure S23. As Figure 2 of the main text but in a high transmission setting corresponding to the first scenario listed in the table on page 27. Annual EIR is 18.0. All $p < 10^{-11}$ when comparing MFT to other strategies. Each boxplot corresponds to 100 simulations.

Varying the distribution of therapies in an MFT strategy.

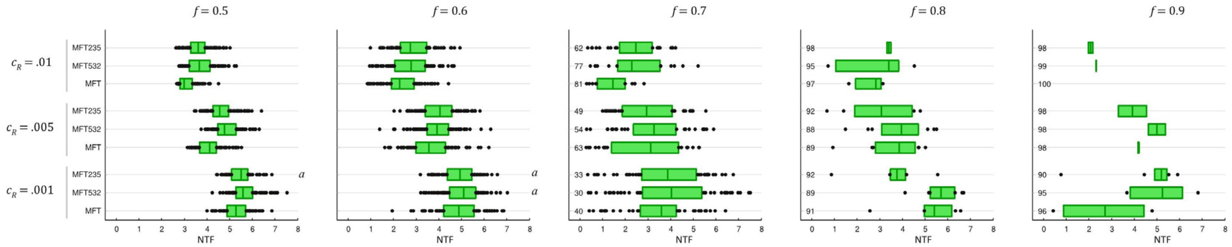

Figure S24. As Figure 2 of the main text, but two new strategies are compared to MFT: MFT235 and MFT532. In MFT235 the proportion of patients that are treated with AL, ASAQ, and DHA-PPQ are 20%, 30%, and 50%, respectively. In MFT532 the proportion of patients that are treated with AL, ASAQ, and DHA-PPQ are 50%, 30%, and 20%, respectively. These simulations were run in a low-transmission scenario, and it does not appear that altering the distribution of therapies according to half-life has a consistent effect on patterns of resistance evolution. One hundred simulations are shown for each scenario. For $f = 0.5$ and $f = 0.6$, MFT has a lower NTF distribution ($p < .004$, except for the comparisons labeled a), but the magnitudes of the differences are small. For $f \geq 0.7$, the number of extinctions is quite high and a statistical comparison of NTF ranges is not done. Mutation rate is highest at intermediate drug concentrations ($k = 4$).

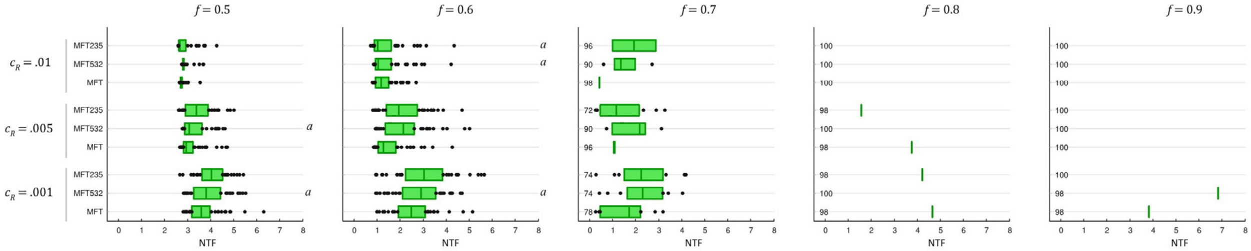

Figure S25. As Figure S24, but the mutation rate is proportional to drug concentration ($k = 0.5$). Fifty simulations are shown for each scenario, but note the small number of simulations plotted for $f \geq 0.7$ as the majority of simulations in these scenarios reached extinction. For $f = 0.5$ and $f = 0.6$, MFT has a lower NTF distribution ($p < .01$, except for the comparisons labeled a), but the magnitudes of the differences are small.

Variation in the surveillance window that determines the 10% treatment threshold.

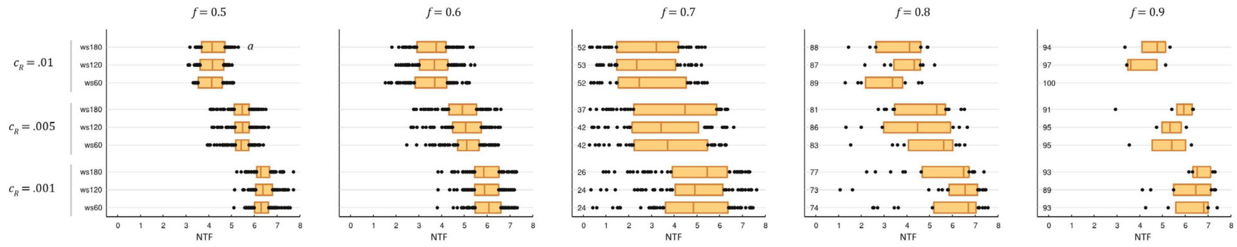

Figure S26. Epidemiological scenario from Figure 2. Sensitivity analysis for the sequential deployment strategy aimed at investigating if the method of computing the 10% treatment-failure threshold will have a large effect on the strategy's long-term outcomes. Normally, we simulate a sequential deployment strategy by assuming that the treatment failure rate is computed by analyzing the successful and failing treatments over the past 60 days ("ws60"). Here, we look at the differences if the past 120 days or past 180 days are used. For $0.5 \leq f \leq 0.7$, there are no statistical differences when "ws60" is compared to either "ws120" or "ws180" (all $p \geq 0.05$) except for the comparison (a) where the 25th quantiles of these distributions are statistically different at $p = 0.022$. For $f \geq 0.8$, the majority of these simulations resulted in extinction (numbers plotted to the left hand side of these panels) and statistical comparison among these distributions is not meaningful. Each boxplot corresponds to 100 model simulations.

Robustness of the biting model

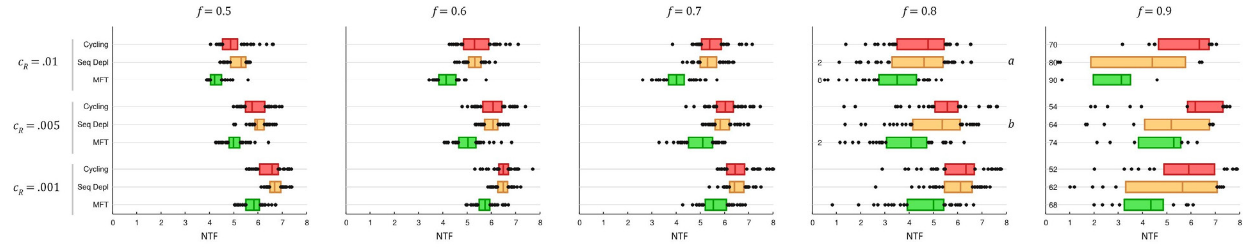

Figure S27. Epidemiological scenario from Figure 2 of main text. Here, the frequency with which a host is bitten by mosquitoes is associated with weight in kilograms. This relationship is motivated by the findings of Port et al [5] and the analysis presented in Smith et al [64]. Weight is taken to be directly proportional to likelihood of biting and age-for-weight measures are taken from WHO [65]. Each boxplot corresponds to 50 simulations, and for $f \leq 0.8$ all $p < 10^{-3}$ when comparing MFT to other strategies except (a) and (b) where the 25th percentiles of these 'Seq Depl' NTF-distributions are not statistically different from MFT. For $f = 0.9$, statistical comparisons among interquartile ranges were not done as the majority behavior in these simulations was extinction.

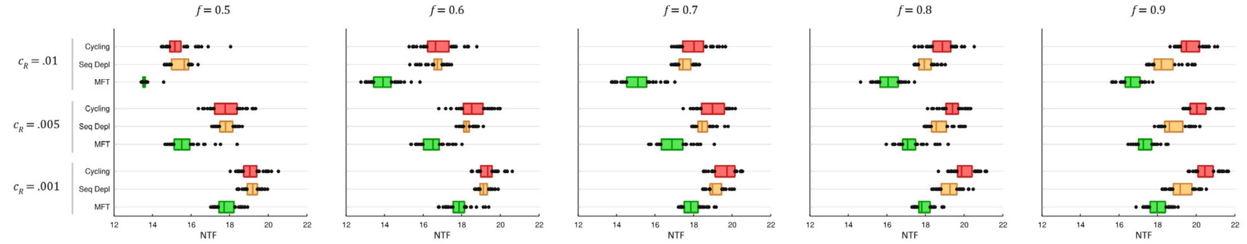

Figure S28. Epidemiological scenario from Figure S22 of this Supplementary Appendix. Here, the probability of an infectious bite causing an infection is proportional to a host's relative level of immunity (which normally depends on age). The data in Hoffman et al [35] suggest that there may be a five-fold or six-fold difference in this probability between children and adults (not accounting for size, weight, or immunity), and in the comparisons presented in this figure we let this infection probability vary from 0.04 to 0.16 according to Prob = 0.04 + 0.12×(1 – ImmuneLevel). The moderate transmission scenario from Figure S22 was chosen for this comparison so that there would be more variation in host immunity in the population. Each boxplot corresponds to 50 simulations, and all $p < 10^{-5}$ when comparing MFT to other strategies.

Small Population Size

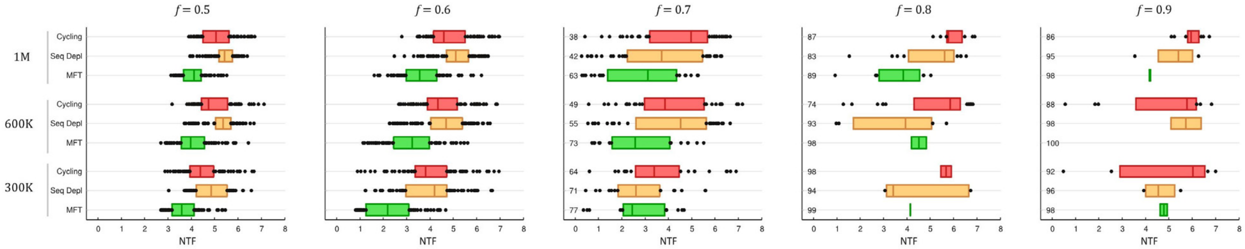

Figure S29. As Figure 2 of the main text (with cost of resistance fixed at c_R = 0.005), and population size is varied here to see if there are any unexpected effects at small population size. The major observed effect is more frequent extinctions observed at small population sizes, which is expected. Each boxplot corresponds to 100 simulations, and the numbers at the left of each panel show what percentage of those simulations reached extinction. For f = 0.5 and f = 0.6, all $p < 10^{-6}$ when comparing MFT to other strategies. For $f \geq 0.7$ the predominant behavior is extinction, and in all cases MFT is associated with more frequent extinction events than either cycling strategy.

19 Comparison to Results in Antao-Hastings (2012)

In a 2012 paper in *Malaria Journal*, Antao and Hastings [66] performed comparisons between MFT and Sequential Deployment strategies. For certain parameter combinations ($0.4 \le f \le 0.6$; MOI = 2 or 4; c_R = 0.10), they observed that MFT strategies resulted in a quicker time to the 10%-treatment-failure milestone and were thus associated with shorter useful therapeutic lives (UTL).

In this section, we attempt to recreate the conditions that led to these outcomes in the Antao-Hastings analysis. However, several important caveats need to be stated and some differences between the models need to be underlined. The differences between the model constructions are substantial, and the comparison presented in this section is approximate.

(*a*) The Antao-Hastings model (called ogaraK) does not explicitly model a population of humans. It only tracks gene frequencies of malaria parasites (as in a traditional population genetics framework). Therefore, some key aspects of malaria epidemiology are missing in this model and are not able to be compared to our model. For example, malaria prevalence cannot be tracked in ogaraK, and as resistance evolves in ogaraK it is not known if prevalence increases or by how much; hence, we cannot compare a prevalence trajectory from our model and the Antao-Hastings model. Likewise, ogaraK does not track clinical/symptomatic cases, age-structure of malaria cases, or MOI distribution, so these general epidemiological measures of malaria cannot be compared.

(*b*) UTLs can be compared between the two models, but NTF values cannot be compared, as a purely population-genetic model cannot track the total number of treatment failures in a single model simulation. We will present the NTF and UTL comparisons from our model below, but it is important to remember that the total number of treatment failures is the more appropriate health outcome measure for policy comparison, as this represents the number of individuals that will receive a failing/ineffective therapy during a fixed time period (in our model, 20 years).

(*c*) OgaraK uses a fixed "generation time" of approximately 50 days, meaning that the model assumes that it takes about 50 days for one malaria infection to generate a second malaria infection. The generation time in our model is variable and dependent on symptoms, age, parasitaemia, biting rate, and current prevalence. The relationship between prevalence and generation time is especially important because at low prevalence the generation time will be longer, resulting in slower evolution in general; at high prevalence, mosquito bites are more common and the generation time is shorter, resulting in a faster process of positive selection or negative selection if either of these is occurring.

(*d*) OgaraK fixes its MOI distribution. The MOI distribution in our model is dependent on prevalence. At high prevalence, individuals get bitten more often by infectious mosquitoes and the number of clonal parasite populations carried by each individual will generally be higher. The ogaraK simulations were run with MOI set to either 2 or 4 for all individuals. Because we cannot calibrate our model so that each host has exactly 2 or 4 clonal parasite populations, we chose prevalence levels where the mean MOI was equal to 2 or 4.

(*e*) There are other model differences that will affect the results. Our simulation has a model of immunity, within-host parasitaemia, immunity-mediated symptoms, immunity-mediated parasitaemia, a transmission cycle that depends on parasite density levels, pharmacokinetics, pharmacodynamics, and biting rate heterogeneity. All of these features will affect the epidemiology and evolution in the simulation to some degree.

First, we attempted to calibrate the transmission setting in our model to mimic the MOI levels in the Antao-Hastings paper. We used these two transmission settings:

Transmission Coefficient (β)	Coefficient of Variation in Biting Rate	Prevalence at Equilibrium	Annual EIR at Equilibrium	Mean of MOI distribution
1.50	2.0	44.0%	94	2.12
8.00	2.0	83.8%	791	4.01

However, after running these at f=0.6 and c_R=0.10, we could not compare results because, in our model, a 60% coverage and a 10% cost of resistance do not generate sufficient selection pressure for rapid resistance evolution (note that both our model and the Antao-Hastings model use a multiplicative fitness model for cost of resistance in multi-drug resistant genotypes). Very little resistance evolution was seen in about 500 model runs, and the useful therapeutic lives of both strategies (MFT and Sequential) were equal to 20 years for a 20-year model simulation. This was not a consequence of the mutation rate or of the introduction/non-introduction of resistant mutants at the beginning of the simulation (we tested both hypotheses). This was simply a consequence of the resistant types experiencing a weak selective environment, which resulted in very slow resistance evolution.

Therefore, we set the cost of resistance to c_R = 0.025 in the MOI=2 scenario, and we set the cost of resistance to c_R = 0.010 in the MOI=4 scenario, in order to get resistance evolution times that were close to those seen in Figures 1 and 3 of the Antao-Hastings paper. Below we present the comparisons between an MFT strategy and a Sequential Deployment strategy under these conditions. For sequential deployment, normally the drugs are deployed as AL first, then AS-AQ, then DHA-PPQ, so that shorter half-life ACTs are used first to minimize drug pressure. For these comparisons, we also tested the inverse order ("Seq Depl Inv"), as the UTLs were quite long in most cases and using the long-half life drug first sometimes minimized prevalence and NTF.

For MOI=2, the comparisons between MFT and Sequential Deployment were:

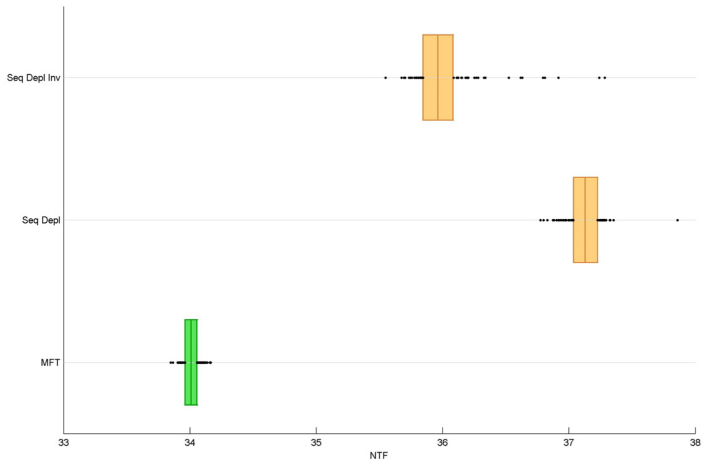

Figure S30: Comparison of MFT and Sequential Deployment (one hundred simulations for each strategy) under the Antao-Hastings scenario of MOI=2. Cost of resistance in this figure is c_R = 0.025. Treatment coverage is f = 0.6. Drug-resistance mutations are more likely at intermediate concentrations (k = 4). All $p < 10^{-15}$ when comparing MFT to other strategies.

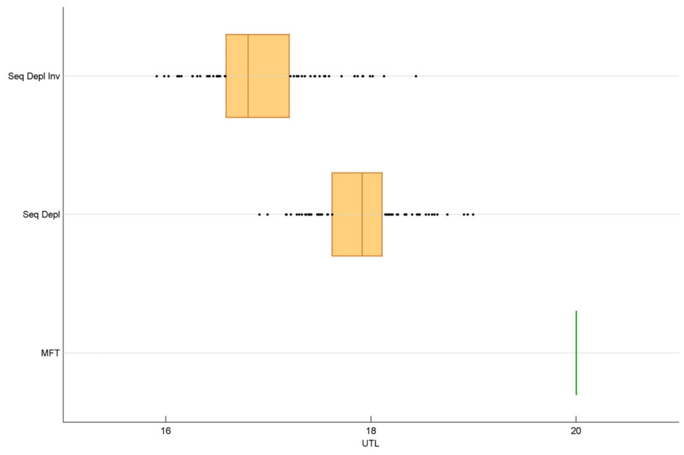

Figure S31: Useful therapeutic lives (UTLs) of drug strategies from Figure S30. All $p < 10^{-15}$ when comparing MFT to other strategies.

For MOI=4, the comparisons between MFT and Sequential Deployment were:

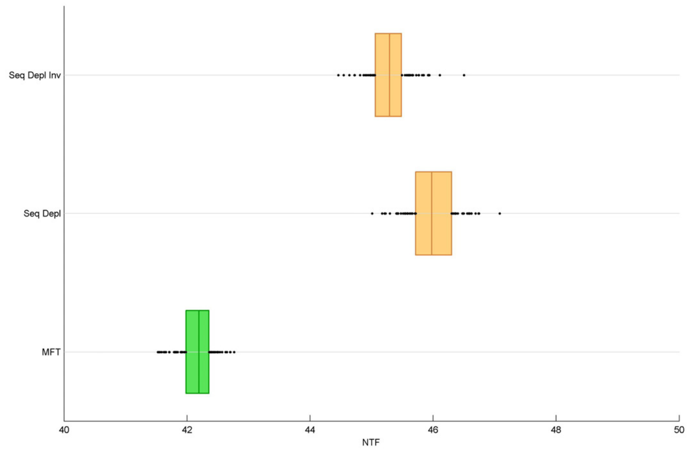

Figure S32: Comparison of MFT and Sequential Deployment (one hundred simulations for each strategy) under the Antao-Hastings scenario of MOI=4. Cost of resistance in this figure is c_R = 0.010. Treatment coverage is f = 0.6. Drug-resistance mutations are more likely at intermediate concentrations (k = 4). All $p < 10^{-15}$ when comparing MFT to other strategies.

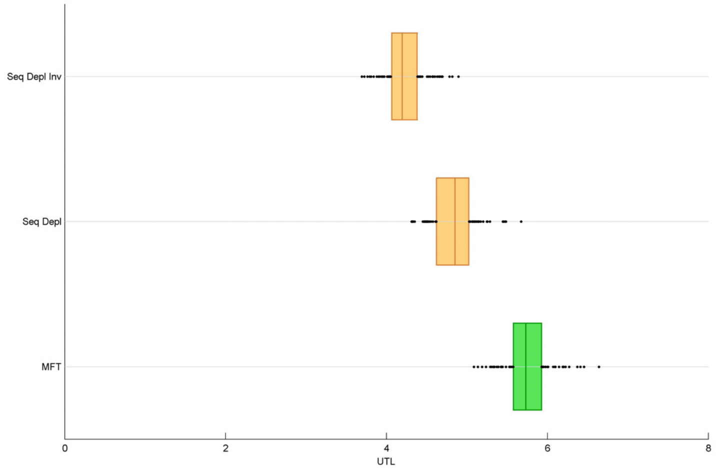

Figure S33: Useful therapeutic lives (UTLs) of drug strategies from Figure S32. All $p < 10^{-15}$ when comparing MFT to other strategies. Drug resistance evolution occurs more quickly in this scenarios than for MOI=2 because the costs of resistance are different for the two figures. No resistance evolution was observed in the MOI=4 scenario when $c_R = 0.025$.

References

1. Andreasen V, Lin J, Levin SA (1997) The dynamics of cocirculating influenza strains conferring partial cross-immunity. J Math Biol 35: 825–842.
2. Smithuis F, Kyaw MK, Phe O, Win T, Aung PP, et al. (2010) Effectiveness of five artemisinin combination regimens with or without primaquine in uncomplicated falciparum malaria : an open-label randomised trial. Lancet 10: 673–681.
3. Smith DL, Dushoff J, Snow RW, Hay SI (2005) The entomological inoculation rate and Plasmodium falciparum infection in African children. Nature 438: 492–495.
4. Ross A, Killeen G, Smith T (2006) Relationships between host infectivity to mosquitoes and asexual parasite density in. Am J Trop Med Hyg 75: 32–37.
5. Port GR, Boreham PFL, Bryan JH (1980) The relationship of host size to feeding by mosquitoes of the Anopheles gambiae Giles complex (Diptera: Culicidae). Bull Entomol Res 70: 133–144.
6. Boreham PF, Lenahan JK, Boulzaguet R, Storey J, Ashkar TS, et al. (1979) Studies on multiple feeding by Anopheles gambiae s.l. in a Sudan savanna area of north Nigeria. Trans R Soc Trop Med Hyg 73: 418–423.
7. Koella JC, Sørensen FL, Anderson RA (1998) The malaria parasite, Plasmodium falciparum, increases the frequency of multiple feeding of its mosquito vector, Anopheles gambiae. Proc R Soc B 265: 763–768.
8. Boni MF, Smith DL, Laminarayan R (2008) Benefits of using multiple first-line therapies against malaria. Proc Natl Acad Sci USA 105: 14216–14221.
9. Eyles DE, Young MD (1951) The duration of untreated or inadequately treated Plasmodium falciparum infections in the human host. J Natl Mal Soc 10: 327.
10. Maire N, Smith T, Owusu-Agyei S, Ross A, Dietz K, et al. (2006) A model for natural immunity to asexual blood stages of Plasmodium falciparum malaria in endemic areas. Am J Trop Med Hyg 75: 19–31.
11. Molineux L, Gramicci G, Molineaux L, Gramiccia G (1980) The Garki Project: research on the epidemiology and control of malaria in the Sudan savanna of West Africa. World Health Organization. Geneva, Switzerland.
12. Lines JD, Wilkes TJ, Lyimo EO (1991) Human malaria infectiousness measured by age-specific sporozoite rates in Anopheles gambiae in Tanzania. Parasitology 102 Pt 2: 167–177.
13. Beier JC (1998) Malaria Parasite Development in Mosquitoes. Annu Rev Entomol 43: 519–543.
14. McKenzie FE, Bossert WH, Bosserrt W (2005) An integrated model of Plasmodium falciparum dynamics. J Theor Biol 232: 411–426.
15. Filipe JA N, Riley EM, Drakeley CJ, Sutherland CJ, Ghani AC (2007) Determination of the processes driving the acquisition of immunity to malaria using a mathematical transmission model. PLoS Comput Biol 3: e255.
16. Mwangi TW, Ross A, Snow RW, Marsh K (2005) Case definitions of clinical malaria under different transmission conditions in Kilifi District, Kenya. J Infect Dis 191: 1932–1939.
17. Saúte F, Aponte J, Almeda J, Ascaso C, Vaz N, et al. (2003) Malaria in southern Mozambique: incidence of clinical malaria in children living in a rural community in Manhiça district. Trans R Soc Trop Med Hyg 97: 655–660.
18. Ghani AC, Sutherland CJ, Riley EM, Drakeley CJ, Griffin JT, et al. (2009) Loss of population levels of immunity to malaria as a result of exposure-reducing interventions: consequences for interpretation of disease trends. PLoS One 4: e4383.
19. Guinovart C, Bassat Q, Sigaúque B, Aide P, Sacarlal J, et al. (2008) Malaria in rural Mozambique. Part I: children attending the outpatient clinic. Mal J 7: 36. doi:10.1186/1475-2875-7-36.
20. Smith T, Killeen G, Lengeler C, Tanner M (2004) Relationships between the outcome of Plasmodium falciparum infection and the intensity of transmission in Africa. Am J Trop Med Hyg 71: 80–86.
21. White NJ (1997) Assessment of the pharmacodynamic properties of antimalarial drugs in vivo. Antimicrob Agents Chemother 41: 1413–1422.
22. Gordeuk V, Thuma P (1993) Iron chelation as a chemotherapeutic strategy for falciparum malaria. Am J Trop Med Hyg 48: 193–197.
23. Hien TT, White NJ (1993) Qinghaosu. Lancet 341: 603–608.
24. Kremsner PG, Winkler S, Brandts C, Graniger W, Bienzle U (1993) Curing of chloroquine-resistant malaria with clindamycin. Am J Trop Med Hyg 49: 650–654.

25. Meek S, Doberstyn E, Gauzere BA, Thanapanich C, Nordlander E, et al. (1986) Treatment of falciparum malaria with quinne and tetracycline or combined mefloquine/sulfadoxine/pyrimethamine on the Thai-Kampuchean border. Am J Trop Med Hyg 35: 246–250.

26. Pukrittayakamee S, Viravan C, Charoenlarp P, Yeamput C, Wilson RJ, et al. (1994) Antimalarial effects of rifampin in Plasmodium vivax malaria. Antimicrob Agents Chemother 38: 511–514.

27. Rieckmann K, Suebsaeng L, Rooney W (1987) Response of Plasmodium falciparum infections to pyrimethamine-sulfadoxine in Thailand. Am J Trop Med Hyg 37: 211–216.

28. Ter Kuile FO, Nosten F, T. Chongsuphajaisiddhi, Dolan G, Luxemburger C, et al. (1993) Halofantrine versus mefloquine in treatment of multidrug-resistant falciparum malaria. Lancet 341: 1044–1049.

29. Ter Kuile FO, Nosten F, Thieren M, Luxemburger C, Edstein MD, et al. (1992) High-dose mefloquine in the treatment of multidrug-resistant falciparum malaria. J Infect Dis 166: 1393–1400.

30. Turaman C, Basco LK, Le Bras J (1992) Evaluating the efficacy of chloroquine in febrile Guinean children infected with Plasmodium falciparum by a simplified in vivo test. Bull World Heal Org 70: 477–480.

31. World Health Organization (2006) Guidelines for the Treatment of Malaria, First Edition. Geneva, Switzerland.

32. Amin AA, Zurovac D, Kangwana BB, Greenfield J, Otieno DN, et al. (2007) The challenges of changing national malaria drug policy to artemisinin-based combinations in Kenya. Mal J 6: 72.

33. Jeffery G (1956) Blood meal volume in Anopheles quadrimaculatus, A. albimanus and Aedes aegypti. Exp Parasitol 375: 371–375.

34. Beier JC, Oster CN, Onyango FK, Bales JD, Sherwood JA, et al. (1994) Plasmodium falciparum incidence relative to entomologic inoculation rates at a site proposed for testing malaria vaccines in western Kenya. Am J Trop Med Hyg 50: 529-536.

35. Hoffman SL, Oster CN, Plowe C V, Woollett GR, Beier JC, et al. (1987) Naturally acquired antibodies to sporozoites do not prevent malaria: vaccine development implications. Science (80-) 237: 639–642.

36. Abdullah S, Adazu K, Masanja H, Diallo D, Ilboudo-Sanogo E, et al. (2007) Patterns of age-specific mortality in children in endemic areas of sub-Saharan Africa. Am J Trop Med Hyg 77: 99-105.

37. Becher H, Kynast-Wolf G, Sié A, Ndugwa R, Ramroth H, et al. (2008) Patterns of malaria: cause-specific and all-cause mortality in a malaria-endemic area of west Africa. Am J Trop Med Hyg 78: 106–113.

38. Goodman C, Coleman P, Mills A (2000) Economic Analysis of Malaria Control in Sub-Saharan Africa. World Health Organization. Geneva, Switzerland.

39. Myint HY, Tipmanee P, Nosten F, Day NP., Pukrittayakamee S, et al. (2004) A systematic overview of published antimalarial drug trials. Trans R Soc Trop Med Hyg 98: 73–81.

40. Nsobya SL, Parikh S, Kironde F, Lubega G, Kamya MR, et al. (2004) Molecular evaluation of the natural history of asymptomatic parasitemia in Ugandan children. J Infect Dis 189: 2220–2226.

41. Kun J, Missinou M, Lell B, Sovric M (2002) New emerging Plasmodium falciparum genotypes in children during the transition phase from asymptomatic parasitemia to malaria. Am J Trop Med Hyg 66: 653–658.

42. Rabarijaona LP, Randrianarivelojosia M, Raharimalala L a, Ratsimbasoa A, Randriamanantena A, et al. (2009) Longitudinal survey of malaria morbidity over 10 years in Saharevo (Madagascar): further lessons for strengthening malaria control. Mal J 8: 190.

43. White NJ, Pukrittayakamee S, Hien TT, Faiz MA, Mokuolu O a, et al. (2013) Malaria. Lancet 383: 723-735.

44. Wongsrichanalai C, Barcus MJ, Muth S, Sutamihardja A, Wernsdorfer WH (2007) A review of malaria diagnostic tools: microscopy and rapid diagnostic test (RDT). Am J Trop Med Hyg 77: 119–127.

45. Hill A (1910) The possible effects of the aggregation of the molecules of haemoglobin on its dissociation curves. J Physiol 40: iv-vii.

46. Kay K, Hastings IM (2013) Improving pharmacokinetic-pharmacodynamic modeling to investigate anti-infective chemotherapy with application to the current generation of antimalarial drugs. PLoS Comput Biol 9: e1003151.

47. World Health Organization (2001) The use of antimalarial drugs. Report of a WHO Informal Consultation, 13-17 November, 2000. World Health Organization. Geneva, Switzerland. http://whqlibdoc.who.int/hq/2001/WHO_CDS_RBM_2001.33.pdf

48. Stepniewska K, Taylor W, Sirima SB, Ouedraogo EB, Ouedraogo A, et al. (n.d.) Population pharmacokinetics of artesunate and amodiaquine in African children. Mal J 8: 200.

49. Byakika-Kibwika P, Lamorde M, Mayanja-Kizza H, Khoo S, Merry C, et al. (2011) Artemether-Lumefantrine Combination Therapy for Treatment of Uncomplicated Malaria: The Potential for Complex Interactions with Antiretroviral Drugs in HIV-Infected Individuals. Malar Res Treat 2011: 703730.

50. Tarning J, Lindegårdh N, Annerberg A, Day NPJ, Ashton M, et al. (2005) Pitfalls in estimating piperaquine elimination. Antimicrob Agents Chemother 49: 5127–5128.

51. Lindblade KA, Steinhardt L, Samuels A, Kachur SP, Slutsker L (2013) The silent threat: asymptomatic parasitemia and malaria transmission. Expert Rev Anti Infect Ther 11: 623–639.

52. Yekutiel P (1960) Problems of epidemiology in malaria eradication. Bull World Heal Org 22: 669–683.

53. Njama-Meya D, Kamya MR, Dorsey G (2004) Asymptomatic parasitaemia as a risk factor for symptomatic malaria in a cohort of Ugandan children. Trop Med Intl Heal 9: 862–868.

54. Cucunubá ZM, Guerra AP, Rahirant SJ, Rivera JA, Cortés LJ, et al. (2008) Asymptomatic Plasmodium spp. infection in Tierralta, Colombia. Mem Inst Oswaldo Cruz 103: 668–673.

55. Beier JC, Killeen GF, Githure JI (1999) Short report: entomologic inoculation rates and Plasmodium falciparum malaria prevalence in Africa. Am J Trop Med Hyg 61: 109–113.

56. Hay SI, Guerra C a, Tatem AJ, Atkinson PM, Snow RW (2005) Urbanization, malaria transmission and disease burden in Africa. Nat Rev Microbiol 3: 81–90.

57. Winskill P, Rowland M, Mtove G, Malima RC, Kirby MJ (2011) Malaria risk factors in north-east Tanzania. Mal J 10: 98.

58. Snow R, Marsh K (2002) The consequences of reducing transmission of Plasmodium falciparum in Africa. Adv Parasitol 52: 235–265.

59. Marsh K, Snow RW (1999) Malaria transmission and morbidity. Parassitologia 41: 241–246.

60. Smith DL, Guerra C a, Snow RW, Hay SI (2007) Standardizing estimates of the Plasmodium falciparum parasite rate. Mal J 6: 131.

61. Owusu-Agyei S, Smith T, Beck H-P, Amenga-Etego L, Felger I (2002) Molecular epidemiology of Plasmodium falciparum infections among asymptomatic inhabitants of a holoendemic malarious area in northern Ghana. Am J Trop Med Hyg 7: 421–428.

62. Arnot D (1998) Unstable malaria in Sudan: the influence of the dry season: clone multiplicity of Plasmodium falciparum infections in individuals exposed to variable levels of disease. Trans R Soc Trop Med Hyg 92: 580-585.

63. Woolhouse MEJ, Dye C, Etard J-F, Smith T, Charlwood JD, et al (1997) Heterogeneities in the transmission of infectious agents: implications for the design of control programs. Proc Natl Acad Sci USA 94: 338–342.

64 . Smith T, Maire N, Dietz K, Killeen GK, Vounatsou P, Molineaux L, Tanner M (2006). Relationship between the Entomologic Inoculation Rate and the Force of Infection for Plasmodium Falciparum Malaria. Am J Trop Med Hyg 75 (Suppl 2): 11–18.

65. World Health Organization. Child growth standards: weight-for-age charts. http://www.who.int/childgrowth/standards/chts_wfa_boys_z/en/. Accessed March 30 2015.

66 Antao T, Hastings I (2012). Policy Options for Deploying Anti-Malarial Drugs in Endemic Countries: A Population Genetics Approach. Mal J 11: 422.
